# *DPYD* genetic polymorphisms in non-European patients with severe fluoropyrimidine-related toxicity: a systematic review

**DOI:** 10.1038/s41416-024-02754-z

**Published:** 2024-06-17

**Authors:** Tsun Ho Chan, J. Eunice Zhang, Munir Pirmohamed

**Affiliations:** Wolfson Centre for Personalised Medicine, Department of Pharmacology and Therapeutics, Institute of Systems, Molecular and Integrative Biology, University of Liverpool, 1-5 Brownlow Street, Liverpool, L69 3GL UK

**Keywords:** Genetics research, Risk factors

## Abstract

**Background:**

Pre-treatment *DPYD* screening is mandated in the UK and EU to reduce the risk of severe and potentially fatal fluoropyrimidine-related toxicity. Four *DPYD* gene variants which are more prominently found in Europeans are tested.

**Methods:**

Our systematic review in patients of non-European ancestry followed PRISMA guidelines to identify relevant articles up to April 2023. Published in silico functional predictions and in vitro functional data were also extracted. We also undertook in silico prediction for all *DPYD* variants identified.

**Results:**

In 32 studies, published between 1998 and 2022, 53 *DPYD* variants were evaluated in patients from 12 countries encompassing 5 ethnic groups: African American, East Asian, Latin American, Middle Eastern, and South Asian. One of the 4 common European *DPYD* variants, c.1905+1G>A, is also present in South Asian, East Asian and Middle Eastern patients with severe fluoropyrimidine-related toxicity. There seems to be relatively strong evidence for the c.557A>G variant, which is found in individuals of African ancestry, but is not currently included in the UK genotyping panel.

**Conclusion:**

Extending UK pre-treatment *DPYD* screening to include variants that are present in some non-European ancestry groups will improve patient safety and reduce race and health inequalities in ethnically diverse societies.

## Introduction

Fluoropyrimidines are antimetabolite chemotherapy drugs comprising the parenterally administered 5-fluorouracil (5-FU) and its prodrugs, capecitabine and tegafur. They are commonly used either as monotherapy or in combination with other antineoplastic agents in neo-adjuvant, adjuvant and palliative settings for a variety of solid tumour types including colorectal, breast, oesophago–gastric and head and neck cancers [1, 2]. 5-FU and capecitabine have been on the World Health Organisation (WHO) Essential Medicines List (EML) since 1977 and 2015, respectively [3, 4]. Annually, over two million patients worldwide and approximately 600,000 patients in Europe receive treatment with fluoropyrimidines [5–7]. Due to a narrow therapeutic index, 10–30% of patients who receive standard fluoropyrimidine doses develop severe toxicity including bone marrow suppression, diarrhoea, mucositis and hand-foot syndrome, usually within the first 1–2 cycles of treatment [8–11]. Severe fluoropyrimidine-related toxicity leads to mortality in approximately 0.5–1% of patients (with up to 5% lethal toxicity reported in elderly patients) [12–16].

Development of toxicity is in part due to inter-individual variability in dihydropyrimidine dehydrogenase (DPD) activity. The first case report of a patient presenting with 5-FU-related severe toxicity due to DPD deficiency was in 1985 [17]. DPD is the primary enzyme responsible for the catabolism and elimination of >80% of the administered 5-FU to the inactive metabolite dihydrofluorouracil (DHFU) [1, 15, 18, 19]. Deficiency of the DPD enzyme, either complete or partial, leads to inadequate clearance of 5-FU which increases drug exposure and accumulation, increasing the risk of severe and sometimes fatal toxicity [20–22]. DPD deficiency can be detected in 39–61% of patients with severe fluoropyrimidine-related toxicity [23]. In individuals of European ancestry, the frequency of partial DPD enzyme deficiency ranges from 3 to 5% while complete DPD enzyme deficiency is less frequent, with an estimated prevalence of 0.1–0.2% [24, 25].

The DPD gene (*DPYD*) is expressed in a wide variety of human tissues; high levels are observed in the liver and peripheral blood mononuclear cells (PBMCs) [26, 27]. Located on chromosome 1p21.3, *DPYD* is a large pharmacogene spanning ~920 kb in length, with 23 relatively small exons (69-961 bp) surrounded by large intronic regions [28, 29]. The coding sequence totals ~3 kb in length and encodes a polypeptide comprising 1,025 amino acid residues [28, 29]. *DPYD* is highly polymorphic: the Genome Aggregation Database (gnomAD v2.1.1) includes 204 synonymous variants and 569 missense variants, 40 of which are predicted to lead to loss of enzymatic function [30].

The latest version of the Clinical Pharmacogenetics Implementation Consortium (CPIC) guideline includes 82 known *DPYD* variants, among which, 21 are considered to have no DPD function and 6 to have diminished DPD function [6]. Prospective genotyping of *DPYD* can identify patients with DPD enzyme deficiency and allow for prophylactic fluoropyrimidine dose adjustments, thereby reducing the likelihood of fluoropyrimidine-related toxicity without compromising the cancer treatment effect [31–35].

In June 2020, the European Medicines Agency (EMA) recommended DPD testing either by phenotyping or genotyping prior to treatment with fluoropyrimidines [36]. In November 2020, the National Health Service (NHS) commissioned *DPYD* genetic testing making this one of the first pharmacogenomic tests to be applied nationally in the UK [37]. A variety of genotyping methods are used by the labs but they all test for the four pathological *DPYD* variants commonly described in Europeans:

- c.1905+1G>A (IVS14+1G>A, rs3918290, DPYD*2A), a splice-site variant causing exon 14 skipping which results in the production of an inactive protein [38, 39];
- c.2846A>T (p.Asp949Val, rs67376798, DPYD*9B), a non-synonymous variant that leads to reduced DPD activity;
- c.1236G>A/HapB3 (p.Glu412Glu, rs56038477), a synonymous variant which tags for c.1129-5923C>G (rs75017182), a deep-intronic splice-site variant causing significant loss of DPD activity, which is in near perfect linkage disequilibrium (LD) with the *DPYD* haplotype HapB3 encompassing three intronic variants (rs56276561, rs6668296, rs115349832); and
- c.1679T>G (p.Ile560Ser, rs55886062, DPYD*13), a missense variant causing decreased DPD activity.

This is because the three key clinical studies which provided evidence for the clinical utility of *DPYD* testing to reduce the incidence of severe fluoropyrimidine-related toxicity were all undertaken in European populations [11, 31, 32]. The minor allele frequencies (MAF) of these four prominent European *DPYD* variants across non-European population groups from the 1000 Genomes Project Phase 3 [40] and gnomAD v3.1.2 and v4.0.0 [41] databases are shown in Supplementary Table 1.

It is known that there are inter-ethnic differences in *DPYD* variant frequency. In fact, several studies have reported the absence of the European *DPYD* variants in populations from East and Southern Africa, namely Somalia, Kenya [42] Zimbabwe [43] and East Asia including China [44] and Japan [45–48]. In addition, variants that are not present in Europeans can have a profound impact in non-European populations, and vice versa [49]. Hence, the testing being undertaken by EU countries and the UK NHS will not identify genetic variants in some non-European populations, who will be treated as wild-type, and given conventional doses of the fluoropyrimidine drugs, with the likelihood of toxicity, and in the worst-case scenario, death. This has the potential to exacerbate health and race inequalities in ethnically diverse societies. Furthermore, it does not help countries where the population is predominantly of non-European ancestry, as *DPYD* genetic testing will not be implemented because of a lack of evidence. It is crucial that all global populations benefit equally from this important genetic test. We have therefore undertaken a systematic review to evaluate *DPYD* genetic variants which have been reported in patients of non-European ancestry who developed severe fluoropyrimidine-related toxicity.

## Methods

### Design and registration

A systematic review was conducted in accordance to the Preferred Reporting Items for Systematic reviews and Meta-Analyses (PRISMA) 2020 guideline [50]. The review protocol was registered in the PROSPERO repository of systematic reviews (registration number CRD42023385227). The EndNote™ X9 software was used to manage all articles (both included and excluded records) throughout the research process.

### Search strategy

A literature search was performed using the MEDLINE (PubMed), Web of Science, Embase (OVID) and Scopus electronic databases to identify relevant articles published prior to 04 April 2023. The search strategy employed a combination of MeSH terms and keywords using the Boolean operators “AND” and “OR”. In addition, syntax adjustments were made appropriate to each database. The search terms used in the MEDLINE (PubMed) search are described in Supplementary Table 2; similar terms were used in the Web of Science, Embase (OVID), and Scopus searches.

### Eligibility criteria

We limited our search to clinical research studies, case series and case reports that genotyped for *DPYD* genetic variants in patients of non-European ancestry who had developed severe (including fatal) fluoropyrimidine-related toxicity after chemotherapy treatment containing 5-FU, capecitabine or tegafur. We accepted the definition of severe toxicities as (1) grade ≥3 severe adverse events according to the Common Terminology Criteria for Adverse Event (CTCAE) [51], (2) grade ≥3 severe adverse events in accordance with the World Health Organization (WHO) [52], and (3) dose-limiting toxicity (DLT) which is defined as pre-specified severe adverse events of grade ≥3 based on the CTCAE classification. To maximise the number of included studies, we also accepted author-defined severity grading of fluoropyrimidine-related toxicities where terms ‘grade ≥3’ or ‘severe’ were used but no classification tool was specified.

Only publications with full-text availability were included. Publications in all languages were assessed with non-English articles translated either via Google Translate or with assistance from colleagues who were native speakers of the foreign language. Authors and titles of conference meeting abstracts were used to check whether full-text articles had been published. Editorials, opinion letters, and unrefereed preprints were not considered.

### Screening process and study selection

After study duplications were removed, T.H.C screened the titles and abstracts of all articles in accordance with the above eligibility criteria to identify the relevant studies for first phase inclusion; irrelevant studies were excluded. In the second phase of the review process, full-text articles of the relevant studies were retrieved, and in-depth full-text screening was carried out. Detailed full-text screening also included the inspection of all cited references. In addition, the reference lists of clinical guidelines, policy statements from regulatory agencies, pertinent narrative and systematic reviews were also screened to check for additional eligible studies. In the situation of any uncertainty during the selection process, the full text was checked and resolved by consensus with J.E.Z.

### Quality assessment

T.H.C and J.E.Z independently assessed the methodological quality of each included study and relied on peer-review to ensure included studies were methodologically sound. The parameters used for assessing clinical research studies, case series and case reports are described in the Supplementary Methods. A formal assessment of the risk of bias was not undertaken.

### Data extraction

Relevant summary and patient-level data from published manuscripts and supplementary materials therein of included studies were independently extracted by T.H.C and J.E.Z. A data extraction form was compiled and data items collected are detailed in the Supplementary Methods. For studies which included patients of European and non-European ancestries, only data reported for non-Europeans were extracted. In instances where information provided in the published manuscript was unclear, we contacted the study authors by email for clarification but amongst the six emails sent out, no response was received, and therefore these 6 articles were excluded. If the exact number for a data item could not be extracted, meticulous estimation was undertaken where possible. All extracted data were presented and compared between T.H.C and J.E.Z, with any disagreements resolved by discussion to reach consensus.

### Data synthesis

Due to the heterogeneity of articles included in this systematic review and the small number of studies conducted in each ethnicity, it was impossible to perform a quantitative analysis, and so the findings are described in a narrative way and data extracted from each article presented in tables, with odds ratios and p-values quoted from the original articles. No meta-analysis was undertaken.

### In silico prediction

In silico prediction was undertaken for all *DPYD* genetic variants evaluated in this systematic review and is described in the Supplementary Methods. The scoring thresholds and software weblinks of the in silico prediction tools used are summarised in Supplementary Table 3.

### Published in silico functional predictions and in vitro functional data

To acquire a more nuanced understanding of the *DPYD* variants identified in our systematic review, published data from previously developed in silico functional prediction models with high accuracy, the DPYD-Varifier [53] and the ADME-optimised Prediction Framework (APF) [54, 55], were extracted (described in Supplementary Methods). In addition, functional data on DPD enzyme activity from in vitro experiments where HEK293T/c17, HEK293-Flp-In and 293FT cells were transiently expressed with *DPYD* variants and treated with either 5-FU or thymine were extracted [42, 45, 56–59].

## Results

### Identification and selection of articles

**Fig. 1:**
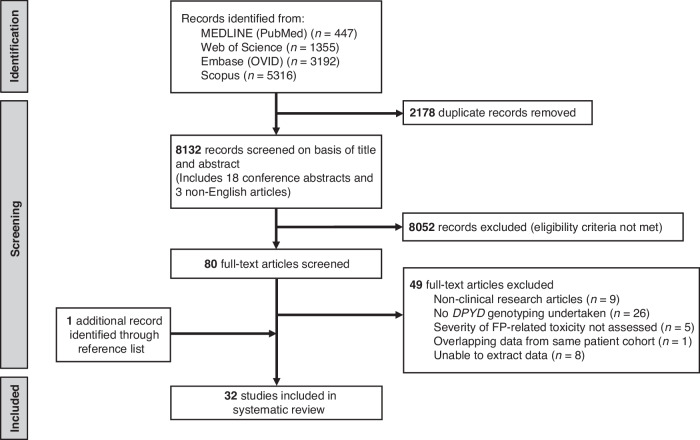
PRISMA flow diagram of study selection. Our search of four electronic databases identified a total of 10310 records, 447 from MEDLINE (PubMed), 1355 from Web of Science, 3192 from Embase (OVID), 5316 from Scopus. After removing 2178 duplicates, 8132 unique records remained which included 18 conference abstracts and 3 non-English articles. Following the title and abstract screening phase, 8052 records that did not meet the inclusion criteria were excluded. Full-text inspection of the remaining 80 articles identified 31 articles that met the eligibility criteria for inclusion. Screening the reference lists of these 31 articles identified one more relevant article, and so 32 articles were finally included in the present systematic review.

A detailed flow diagram showing the identification and selection process for study inclusion, according to the PRISMA statement, is depicted in Fig. 1. All articles included were in English; none of the non-English articles met the criteria for inclusion.

### Characteristics of included articles

Table 1 details the 32 included articles and a summary breakdown of the characteristics is provided in Supplementary Table 4. All articles were published between December 1998 and December 2022. Two studies were case series, 10 studies were case reports and 20 were cohort studies with an equal split between prospective and retrospective study designs. Patients were from 12 countries encompassing 5 ethnic groups: African American (United States), East Asian (China, Japan, Korea, Thailand), Latin American (Chile), Middle Eastern (Jordan, Lebanon, Saudi Arabia, Tunisia), and South Asian (Bangladesh, India, United States).

**Table 1:** Characteristics of included studies.

Authors, year	Study design	Ethnic population, Ethnic origin, Country	Severe FP-related toxicity patients/Total patients (n)	Gender	Age (years)	Cancer type	Chemotherapy regimen^abc^	Severe toxicity grading tool	Severe (including fatal) FP-related toxicity	*DPYD* variants identified^ɸ^	*DPYD* genetic testing method	FP dose modification or discontinuation	Ref
Saif et al., 2014	Case study	African American, African American, USA	1/1	Female	60	Colon cancer	5-FU-based	Unreported, used term ‘severe'	Pancytopenia, Mucositis *Death potentially due to severe 5-FU-related toxicities	n.688+20094C>T c.85T>C, p.Cys29Arg, *9A c.557A>G, p.Tyr186Cys c.680+139G>A c.681-29G>T c.763-118A>G c.1906-123C>A c.1974+75T>C c.2766+37T>C c.2908-69A>G c.*768G>A	Sanger sequencing of all 23 exons in *DPYD*	Yes	[60]
Maharjan et al., 2019	Cohort retrospective	Mixture of ethnicities ^d^, American, USA	~22/35^d^	~55% Male	21–90	GI malignancies (38% Colon cancer, 32% Rectal cancer, 10% Pancreatic cancer, 6% Gastric cancer, 14% Other)	70% Fluorouracil-based, 30% Capecitabine-based	CTCAE version 5.0	Neutropenia, Diarrhoea, Mucositis, Vomiting/Nausea, Skin toxicity, Neurotoxicity	c.85T>C, p.Cys29 Arg, *9A	Candidate genotyping of 5 selected variants c.-1590T>C, c.85T>C, c.1679T>G, c.1905+1G>A, c.2846A>T	No	[64]
Leung et al., 2021	Case study	African American, African American, USA	1/1	Female	52	Splenic flexure colon cancer	5-FU-based	Unreported, used term ‘grade 4'	Neutropenia	c.557A>G, p.Tyr186Cys	Sequencing of exons and intron-exon boundaries	Yes	[62]
Sissung et al., 2021	Case study	African American, African American, USA	1/1	Female	63	Metastatic colon cancer	5-FU-based	Unreported, used term ‘severe'	Pancytopenia	c.40-3123T>A c.85T>C, p.Cys29Arg, *9A c.299_302del^§^, p.Phe100fs, *7 c.557A>G, p.Tyr186Cys c.851-18271A>G c.1340-11501T>C c.1898delC, p.Pro633fs, *3 c.1906-28506C>G c.*5132C>T c.*21528C>T	DMET Plus and Pharmacoscan arrays	Yes	[63]
Zhang et al., 2013	Cohort prospective	East Asia, Chinese, China	14/60	57% Male	40–68	Colorectal cancer	5-FU-based	WHO	Bone marrow toxicity, Gastrointestinal toxicity	c.85T>C, p.Cys29Arg, *9A c.464T>A, p.Leu155Ter c.2194G>A, p.Val732Ile, *6	TaqMan genotyping of 5 selected variants c.85T>C, c.464T>A, c.1156G>T, c.1905+1G>A, c.2194G>A	No	[71]
Sun et al., 2014	Cohort prospective	East Asia, Chinese, China	~65/100	57% Male	31–71	Colon cancer	5-FU-based	WHO	Myelosuppression, Diarrhoea, Mucositis, Gastrointestinal toxicity, Hand-foot syndrome	c.85T>C, p.Cys29Arg, *9A c.1627A>G, p.Ile543Val, *5 c.1905+1G>A, *2A	High resolution melting of 3 selected variants c.85T>C, c.1627A>G, c.1905+1G>A	No	[70]
Liu et al., 2017	Cohort retrospective	East Asia, Chinese, China	~139/661	61% Male	47–-63	Metastatic colorectal cancer	5-FU-based, Capecitabine-based, Tegafur-based or Irinotecan monotherapy	CTCAE version 4.0	Neutropenia, Diarrhoea	c.1627A>G, p.Ile543Val, *5 c.1896T>C, p.Phe632Phe	Sanger sequencing of 3 selected variants c.1627A>G, c.1896T>C, c.1905+1G>A	No	[68]
Nie et al., 2019	Cohort prospective	East Asia, Chinese, China	~75/100	56% Male	51–77	Advanced colorectal cancer	5-FU-based	WHO	Myelosuppression, Mucosal damage, Gastrointestinal toxicity, Liver function damage	c.85T>C, p.Cys29Arg, *9A c.1627A>G, p.Ile543Val, *5	Sanger sequencing of 2 selected variants c.85T>C, c.1627A>G	No	[69]
Deng et al., 2020	Cohort retrospective	East Asia, Chinese, China	~72/104	46% Male	25–78	Colorectal cancer	5-FU-based, Capecitabine-based or Oxaliplatin-based	CTCAE version 3.0	Anaemia, Leukopenia, Neutropenia, Thrombocytopenia, Mucositis, Vomiting, Diarrhoea, Hand-foot syndrome, Skin ulceration	c.85T>C, p.Cys29Arg, *9A c.1627A>G, p.Ile543Val, *5	Sanger sequencing of 3 selected variants c.85T>C, c.1627A>G, c.1905+1G>A	No	[67]
Shao et al., 2022	Case study	East Asia, Chinese, China	1/1	Male	68	Rectal cancer	Capecitabine-based	CTCAE version 5.0	Diarrhoea	c.85T>C, p.Cys29Arg, *9A c.1627A>G, p.Ile543Val, *5	Whole exome sequencing	Yes	[72]
Tong et al., 2018	Case study	East Asia, Chinese, Hong Kong	1/1	Female	49	Sigmoid colon carcinoma	Capecitabine-based and 5-FU-based	CTCAE version 4.0	Bone marrow toxicity, Diarrhoea	c.321+2T>C	Sanger sequencing of all 23 exons in *DPYD*	Yes	[73]
Kouwaki et al., 1998	Case study	East Asia, Japanese, Japan	1/1	Female	57	Breast cancer	5-FU-based	WHO	Leukopenia, Thrombocytopenia, Mucositis	c.62G>A, p.Arg21Gln c.1003G>T, p.Val335Leu, *11 c.1156G>T, p.Glu386Ter, *12	PCR-RFLP of exons 2 and 11, Sanger sequencing of exon 10	Yes	[74]
Yoshida et al., 2015	Case study	East Asia, Japanese, Japan	1/1	Male	73	Jejunal cancer	Capecitabine-based	Unreported, used term ‘grade 4'	Leukopenia, Neutropenia, Thrombocytopenia	c.1156G>T, p.Glu386Ter, *12	Sanger sequencing of all 23 exons in *DPYD*	Yes	[75]
Ishiguro et al., 2020	Case study	East Asia, Japanese, Japan	1/1	Male	63	Stomach adenocarcinoma	Capecitabine-based	CTCAE version 4.0	Febrile neutropenia, Diarrhoea, Oral mucositis, Renal dysfunction *Death due to gastric cancer progression following patient’s decision to discontinue chemotherapy	c.1615G>C, p.Gly539Arg c.1627A>G, p.Ile543Val, *5 c.1740+40A>G c.1740+39C>T c.1896T>C, p.Phe632Phe c.1974+75T>C IVS22+585C>T IVS23-69A>G	Sanger sequencing of all 23 exons in *DPYD*	Yes	[61]
Yokoi et al., 2020	Cohort retrospective	East Asia, Japanese, Japan	55/301	44% Male	22–81	69% Colorectal cancer, 20% Stomach cancer, 11% Other	5-FU-based	CTCAE version 4.0	Neutropenia, Diarrhoea, Vomiting Nausea, Oral mucositis	c.85T>C, p.Cys29Arg, *9A c.496A>G, p.Met166Val c.596G>A, p.Ser199Asn c.733A>G, p.Ile245Val c.1156G>T, p.Glu386Ter, *12 c.1627A>G, p.Ile543Val, *5 c.1712C>A, p.Ala571Asp c.1863G>T, p.Trp621Cys c.2194G>A, p.Val732Ile, *6 c.2303C>A, p.Thr768Lys	NGS of exons and flanking introns	No	[48]
Kanai et al., 2022	Cohort retrospective	East Asia, Japanese, Japan	~495/1364	U/R	U/R	Colon cancer	5-FU-based or Capecitabine-based	CTCAE version 3.0 and 4.0	Neutropenia, Diarrhoea, Mucositis, Hand-foot syndrome	c.85T>C, p.Cys29Arg, *9A c.451A>G, p.Asn151Asp c.496A>G, p.Met166Val c.1003G>T, p.Val335Leu, *11 c.1627A>G, p.Ile543Val, *5 c.2194G>A, p.Val732Ile, *6 c.2303C>A, p.Thr768Lys	Genome-wide genotyping	No	[46]
Cho et al., 2007	Cohort retrospective	East Asia, Korean, Korea	21/21	43% Male	31–71	Colorectal cancer	5-FU-based	CTCAE version 2.0	Neutropenia, Stomatitis, Diarrhoea, Vomiting/Nausea, Fatigue, Fever	c.85T>C, p.Cys29Arg, *9A c.496A>G, p.Met166Val c.1129-15T>C c.1525-11G>A c.1525-9A>G c.1627A>G, p.Ile543Val, *5 c.1737T>C, p.Asp579Asp c.1740+39C>T c.1774C>T, p.Arg592Trp c.1896T>C, p.Phe632Phe	Sanger sequencing of exons and flanking introns	No	[76]
Sirachainan et al., 2012	Cohort retrospective	East Asia, Thai, Thailand	76/116	U/R	U/R	52% Breast cancer, 35% Gastrointestinal tract cancer, 12% Head and neck cancer, 1% Squamous cell cancer	5-FU-based	Unreported, used term ‘grade ≥3'	Neutropenia	c.967G>A, p.Ala323Thr c.1236G>A/HapB3, p.Glu412Glu c.1627A>G, p.Ile543Val, *5 c.1774C>T, p.Arg592Trp c.1896T>C, p.Phe632Phe c.1905+1G>A, *2A	Sanger sequencing of exons 1, 8, 10, 11, 13, 14 and 17	No	[77]
Cordova-Delgado et al., 2021	Cohort retrospective	Latin American, Chilean, Chile	32/93	59% Male	28–77	Gastric cancer	84% 5-FU-based, 16% Capecitabine-based	CTCAE version 4.0	Anaemia, Neutropenia, Febrile Neutropenia, Diarrhoea, Vomiting/Nausea, Stomatitis, Hand-foot syndrome, Peripheral neuropathy	c.85T>C, p.Cys29Arg, *9A c.496A>G, p.Met166Val c.1627A>G, p.Ile543Val, *5	TaqMan genotyping of 4 selected variants c.85T>C, c.496A>G, c.1627A>G, c.1679T>G (absent)	No	[79]
Almashagbah et al., 2022	Cohort prospective	Middle East, Jordanian, Jordan	44/80	53% Male	~48 (mean)	Colorectal cancer	5-FU-based	Dose-limiting toxicity	Neutropenia, Thrombocytopenia, Haemorrhage, Thrombosis, Diarrhoea, Neurotoxicity, Proteinuria, Hypertension	c.85T>C, p.Cys29Arg, *9A g.97515583_97515584insA c.1740+40A>G c.1740+39C>T	Sanger sequencing of exons 2, 4, 13, 22, intron 13 and exon-intron boundaries	No	[82]
Mukherji et al., 2019	Case study	Middle East, Lebanese, Lebanon	1/1	Female	59	Metastatic pancreatic cancer	5-FU-based	Unreported, used term ‘grade 4'	Mucositis	c.1601G>A, p.Ser534Asn, *4 c.1905+1G>A, *2A c.2194G>A, p.Val732Ile, *6	NGS of exons and highly conserved intron-exon splice junctions	Yes	[83]
Bukhari et al., 2021	Case series	Middle East, Saudi Arabian, Saudi Arabia	3/3	33% Male	64–66	Colorectal cancer	Capecitabine-based and/or 5-FU-based	CTCAE (version unreported)	Neutropenia, Pancytopenia, Diarrhoea, Mucositis, Fatigue	c.257C>T, p.Pro86Leu c.1601G>A, p.Ser534Asn, *4 c.2434G>A, p.Val812Ile	NGS of exons and eight selected intron-exon boundaries	Yes	[84]
Ben Fredj et al., 2009	Cohort prospective	Middle East, Tunisian, Tunisia	2/9	33% Male	25–79	Advanced colorectal cancer	5-FU-based	Unreported, used term ‘grade 3'	Alopecia, Leukopenia, Diarrhoea	c.85T>C, p.Cys29Arg, *9A c.496A>G, p.Met166Val c.1129-15T>C c.1601G>A, p.Ser534Asn, *4 c.1627A>G, p.Ile543Val, *5	DHPLC and Sanger sequencing	No	[80]
Khalij et al., 2022	Cohort prospective	Middle East, Tunisian, Tunisia	~20/66	U/R	~55 (mean)	Colorectal cancer	5-FU or Capecitabine-based	CTCAE version 3.0	Haematotoxicity, Mucositis, Neurotoxicity	c.85T>C, p.Cys29Arg, *9A c.1679T>G, p.Ile560Ser, *13	PCR-RFLP of 5 selected variants including c.85T>C, c.496A>G, c.1679T>G, c.1905+1G>A, c.483+18G>A	No	[81]
Nahid et al., 2018	Cohort prospective	South Asia, Bangladeshi, Bangladesh	78/161	55% Male	25–75	Colorectal cancer	5-FU-based	CTCAE version 3.0	Anaemia, Leukopenia, Neutropenia, Thrombocytopenia, Diarrhoea, Mucositis, Vomiting/Nausea, Dermatological toxicity	c.1905+1G>A, *2A	PCR-RFLP of c.1905+1G>A	No	[92]
Dhawan et al., 2013	Cohort prospective	South Asia, Indian, India	2/23	Male	18–60	Head and neck cancer	5-FU-based	CTCAE version 3.0	Not specified, referred to as grade 3–4 toxicity	c.85T>C, p.Cys29Arg, *9A c.1905+1G>A, *2A	Allele-specific multiplex PCR and long-range PCR of 4 selected variants c.85T>C, c.1905+1G>A, c.2194G>A, c.2846A>T	No	[85]
Rastogi et al., 2014	Case series	South Asia, Indian, India	3/3	66% Male	44–65	Colorectal cancer	Capecitabine-based and Tegafur-based	Unreported, used term ‘grade ≥3'	Neutropenia, Febrile neutropenia, Thrombocytopenia, Diarrhoea, Mucositis, Hand-foot syndrome	c.496A>G, p.Met166Val c.1627A>G, p.Ile543Val, *5 c.1905+1G>A, *2A	Candidate genotyping	Yes	[90]
Patil et al., 2016	Cohort prospective	South Asia, Indian, India	10/34	74% Male	21–59	Advanced head and neck cancer	5-FU-based	Unreported, used term ‘grade ≥3'	Diarrhoea, Mucositis	c.85T>C, p.Cys29Arg, *9A c.496A>G, p.Met166Val c.1601G>A, p.Ser534Asn, *4 c.1627A>G, p.Ile543Val, *5 c.2194G>A, p.Val732Ile, *6	PCR-sequencing of 11 selected variants including c.85T>C, c.496A>G, c.1601G>A, c.1627A>G, c.1905+1G>A, c.2194G>A, c.2846A>T	Yes	[87]
Sahu et al., 2016	Cohort prospective	South Asia, Indian, India	28/506	71% Male	26–67	70% Colorectal cancer, 29% Stomach cancer, 1% Gallbladder cancer	Capecitabine-based	CTCAE (version unreported)	Myelosuppression, Diarrhoea, Mucositis, Hand-foot syndrome	c.85T>C, p.Cys29Arg, *9A c.496A>G, p.Met166Val c.1627A>G, p.Ile543Val, *5 c.1905+1G>A, *2A c.2194G>A, p.Val732Ile, *6	PCR-sequencing of 11 selected variants including c.85T>C, c.496A>G, c.1601G>A, c.1627A>G, c.1905+1G>A, c.2194G>A, c.2846A>T	Yes	[88]
Hariprakash et al., 2018	Cohort retrospective	South Asia, Indian, India	~23/110	68% Male	15–82	70% Colorectal cancer, 8% Stomach cancer, 6% Oesophageal cancer, 5% Gastro-oesophageal junction cancer, 10% Other	55% Capecitabine-based, 45% 5-FU-based	Unreported, used term ‘grade ≥3'	Diarrhoea, Hand-foot syndrome	c.496A>G, p.Met166Val c.1905+1G>A, *2A	Sanger sequencing of 15 selected variants including c.496A>G, c.557A>G (absent), c.1905+1G>A, c.1679T>G, c.2846A>T	No	[86]
Vinin et al., 2018	Cohort retrospective	South Asia, Indian, India	24/40	65% Male	24–77	72.5% Colorectal cancer, 17.5% Stomach cancer, 5% Breast cancer, 2.5% Tongue cancer 2.5% Other	71% Capecitabine-based, 29% 5-FU-based	Unreported, used term ‘grade ≥3'	Diarrhoea, Neutropenia, Thrombocytopenia, Hand-foot syndrome, Mucositis, Electrolyte imbalance, Fatigue	c.85T>C, p.Cys29Arg, *9A c.496A>G, p.Met166Val c.1627A>G, p.Ile543Val, *5 c.1905+1G>A, *2A c.2194G>A, p.Val732Ile, *6	PCR-sequencing: region/variant unspecified	No	[89]
Ly et al., 2020	Case study	South Asia, Indian, USA	1/1	Female	59	Metastatic colon cancer	Capecitabine-based and 5-FU-based	Unreported, used term ‘grade 4’	Mucositis	c.704G>A, p.Arg235Gln	Candidate genotyping and whole genome sequencing	Yes	[91]

*5-FU* 5-fluorouracil, *CTCAE* Common Terminology Criteria for Adverse Events, *DPYD* Dihydropyrimidine dehydrogenase gene, *FP* Fluoropyrimidine, *GI* gastrointestinal, *U/R* unreported, *WHO* World Health Organisation.

^a^5-FU-based regimens include 5-FU + carboplatin; 5-FU + carboplatin + docetaxel; *CF* 5-FU + cisplatin; 5-FU + cisplatin + cetuximab; 5-FU + cisplatin + docetaxel; 5-FU + cisplatin + epirubicin; 5-FU + cisplatin + etoposide; 5-FU + oxaliplatin; *FOLFOX/FOLFOX4/mFOLFOX/mFOLFOX6* 5-FU + oxaliplatin + leucovorin; *FLOT* 5-FU + oxaliplatin + leucovorin + docetaxel; *FOLFIRINOX/FOLFOXIRI/FOLFOXIRI* + *α* 5-FU + oxaliplatin + leucovorin + irinotecan; FOLFOX + panitumumab; 5-FU + irinotecan; *FOLFIRI/IFL* 5-FU + irinotecan + leucovorin; 5-FU + leucovorin; 5-FU + leucovorin + radiation; 5-FU + docetaxel + gemcitabine; *CMF* 5-FU + cyclophosphamide + methotrexate; *FAC* 5-FU + cyclophosphamide + adriamycin; *CEF* 5-FU + cyclophosphamide + epi-adriamycin; *5’DFUR* + *TOR* 5’deoxy-5-fluoro-uridine + toremifene citrate.

^b^Capecitabine-based regimens include Capecitabine + cisplatin + trastuzumab; *CAPEOX/CAPOX/XELOX* Capecitabine + oxaliplatin; XELOX + bevacizumab; *DOX* Capecitabine + oxaliplatin + docetaxel; *EOX* Capecitabine + oxaliplatin + epirubicin; Capecitabine + radiation; (1) CAPOX, (2) capecitabine monotherapy.

^c^Tegafur-based regimens include Tegafur + irinotecan; Tegafur + irinotecan + gimeracil + oteracil; Tegafur + uracil + oxaliplatin.

^d^The cohort study by Maharjan et al. 2019 [63] included patients of a range of ethnicities (Caucasian, African American, Asian, Hispanic, and Native American). Only data from patients of African American ancestry with severe fluoropyrimidine-related toxicity (grade ≥ 3) were extracted and presented in this table.

^ɸ^Reference sequences NM_000110.4 and NP_000101.2 were used for Human Genome Variation Society (HGVS) nomenclatures.

^§^c.299_302del is also known as c.295_298delTCAT (PharmGKB).

Heterogeneity was present across the 32 articles included. Various classification tools and different versions of the same classification tool were used to define the severity of fluoropyrimidine-related toxicity; 15 used CTCAE (one used version 2.0, four used version 3.0, one used version 3.0 and 4.0, five used version 4.0, two used version 5.0, two did not specify the version used), 4 used WHO, 1 used DLT with grade 4 specified. Twelve publications did not report the classification tool used but used the terms ‘grade 3’ (*n* = 1), ‘grade ≥3’ (*n* = 5), ‘grade 4’ (*n* = 4), and ‘severe’ (*n* = 2); results of laboratory blood tests were reported in 6 of these publications (see Supplementary Table 5) which will be classified as grade ≥3 toxicity based on CTCAE version 5.0. Multiple *DPYD* genetic testing methods were employed across the studies ranging from candidate genotyping (*n* = 10), targeted variant sequencing (*n* = 9), targeted variant genotyping and sequencing (*n* = 2), *DPYD* exome sequencing (*n* = 4), sequencing of *DPYD* exome and flanking introns (*n* = 5), to whole exome/genome sequencing (n = 2). Of the 20 cohort studies included, 18 conducted statistical tests for association but a variety of comparisons were made including grade ≥3 versus grade ≤2 toxicity (*n* = 10), all grades of toxicity versus no toxicity (*n* = 4), grade ≥3 toxicity versus healthy (*n* = 1), standard fluoropyrimidine dose versus reduced fluoropyrimidine dose (*n* = 2), and change in absolute neutrophil count, haematocrit, platelet and percentage of neutrophil (*n* = 1).

### Patient characteristics

A summary of the patient characteristics is presented in Table 2. A total of 1313 patients were included across the 32 studies. Their age ranged between 15 and 90 years, and slightly more men than women were enroled in most studies. The most common type of tumour was colorectal cancer and most patients received either 5-FU or capecitabine based combination chemotherapy treatment that included oxaliplatin. All patients were reported to have experienced grade 3 or higher fluoropyrimidine-related toxicities (as defined above). Clinical manifestations included haematological, gastrointestinal, dermatological, neurological, hepatic, and renal toxicities, with many with myelosuppression, neutropenia, diarrhoea, mucositis and hand–foot syndrome. Two fatalities were reported, one potentially due to severe fluoropyrimidine-related toxicity [60] and the other due to cancer progression following discontinuation of chemotherapy [61].

**Table 2:** Patient characteristics.

	All	African American	East Asian	Latin American	Middle Eastern	South Asian
Patients (n)^a^	1313	25	1017	32	70	169
Age range (years)	15–90	21–90	22–81	28–77	25–79	15–82
Gender (% Male)	56	48	54	59	46	65
Cancer type (n)						
Gastrointestinal	1240	25	961	32	70	152
Colorectal	1138	18	921	0	69	130
Stomach	59	1	12	32	0	14
Other^b^	13	6	1	0	1	5
Breast	41	0	40	0	0	1
Head and neck	22	0	9	0	0	13
Squamous cell carcinoma and other unspecified cancers	7	0	7	0	0	3
Chemotherapy regimen (n)						
*5-FU based*	868	18	660	27	61	102
5-FU monotherapy	45	0	1	0	44	0
With platinum	579	3	484	26	8	58
Carboplatin^c^	2	0	2	0	0	0
Cisplatin^d^	39	0	17	8	0	14
Oxaliplatin^e^	538	3	465	18	8	44
With irinotecan^f^	95	0	44	0	7	44
Other^g^	84	0	81	1	2	0
Unreported	65	15	50	0	0	0
* Capecitabine based*	392	7	314	5	8	58
Capecitabine monotherapy	6	0	0	0	0	6
With cisplatin^h^	1	0	1	0	0	0
With oxaliplatin^j^	301	0	246	5	7	43
With irinotecan (CAPIRI)	46	0	45	0	0	1
With radiotherapy	9	0	0	0	1	8
Unreported	29	7	22	0	0	0
*Tegafur based*^m^	40	0	40	0	0	0
*Combination*^n^	16	0	4	0	2	10
Severe toxicity manifestations events (n)						
Haematological^q^	928	11	705	17	68	127
Gastrointestinal^r^	715	36	438	13	26	202
Dermatological^s^	215	8	147	8	1	51
Neurotoxicity^t^	19	4	3	2	8	2
Hepatotoxicity^u^	13	0	13	0	0	0
Renal toxicity^v^	2	0	1	0	1	0
Other^w^	7	0	2	0	3	2
Unspecified	2	0	0	0	0	2
Fatality (n)	2	1^x^	1^y^	0	0	0
Fluoropyrimidine dose modification (n)	8	1	2	0	1	4
Fluoropyrimidine discontinuation (n)	6	1	2	0	2	1
*DPYD* variants (n)	53	19	30	3	13	7
*DPYD* haplotypes (n)	28	2	17	4	2	5
DPD activity (n)						
PBMCs	3	0	3	0	0	0
Plasma UH2/U ratio	2	0	1	0	1	0

*5-FU* 5-fluorouracil, *DPYD* Dihydropyrimidine dehydrogenase gene, *DPD* Dihydropyrimidine dehydrogenase, *PBMCs* peripheral blood mononuclear cells, *UH2/U* dihydrouracil/uracil plasma ratio.

^a^Number of patients who developed fluoropyrimidine-related severe toxicity (grade ≥3).

^b^Other gastrointestinal cancers include oesophageal cancer, gastro-oesophageal cancer, pancreatic cancer, gall bladder cancer, jejunal cancer, small bowel cancer, appendix carcinoma.

^c^Includes: 5-FU + carboplatin; 5-FU + carboplatin + docetaxel.

^d^Includes: *CF* 5-FU + cisplatin; 5-FU + cisplatin + cetuximab; 5-FU + cisplatin + docetaxel; 5-FU + cisplatin + epirubicin; 5-FU + cisplatin + etoposide.

^e^Includes: 5-FU + oxaliplatin; *FOLFOX/FOLFOX4/mFOLFOX/mFOLFOX6* 5-FU + oxaliplatin + leucovorin; *FLOT* 5-FU + oxaliplatin + leucovorin + docetaxel; *FOLFIRINOX/FOLFOXIRI/FOLFOXIRI* + *α* 5-FU + oxaliplatin + leucovorin + irinotecan; FOLFOX + panitumumab.

^f^Includes: 5-FU + irinotecan; *FOLFIRI/IFL* 5-FU + irinotecan + leucovorin.

^g^Includes: 5-FU + leucovorin; 5-FU + leucovorin + radiation; 5-FU + docetaxel + gemcitabine; *CMF* 5-FU + cyclophosphamide + methotrexate; *FAC* 5-FU + cyclophosphamide + adriamycin.

^h^Includes: Capecitabine + cisplatin + trastuzumab.

^j^Includes: *CAPEOX/CAPOX/XELOX* Capecitabine + oxaliplatin; XELOX + bevacizumab; *DOX* Capecitabine + oxaliplatin + docetaxel; *EOX* Capecitabine + oxaliplatin + epirubicin.

^m^Includes: Tegafur + irinotecan; Tegafur + irinotecan + gimeracil + oteracil.

^n^Includes: (1) Capecitabine, (2) 5-FU; (1) XELOX, (2) FOLFOX6; (1) CAPOX, (2) FOLFOX; (1) Capecitabine + oxaliplatin + bevacizumab, (2) mFOLFOX; (1) CAPOX, (2) Tegafur + uracil + oxaliplatin; (1) CAPOX, (2) Capecitabine monotherapy; (1) *CEF* 5-FU + cyclophosphamide + epi-adriamycin, (2) *5’DFUR* + *TOR* 5’deoxy-5-fluoro-uridine + toremifene citrate.

^q^Haematological toxicity includes myelosuppression/bone marrow toxicity, neutropenia, febrile neutropenia, leukopenia, thrombocytopenia, pancytopenia, anaemia, haemorrhage, and thrombosis.

^r^Gastrointestinal toxicity includes diarrhoea, mucositis, vomiting, and nausea.

^s^Dermatological toxicity includes hand-foot syndrome, stomatitis/oral mucositis/mucosal damage, skin ulceration, and alopecia.

^t^Neurotoxicity includes peripheral neuropathy and encephalopathy.

^u^Hepatoxicity includes liver function damage.

^v^Renal toxicity includes renal dysfunction and proteinuria.

^w^Other toxicities include fatigue and fever.

^x^Fatality potentially due to severe 5-FU-related toxicity.

^y^Fatality due to cancer progression following discontinuation of chemotherapy at patient’s discretion.

### *DPYD* genetic variants, haplotypes and in silico predictions

**Fig. 2:**
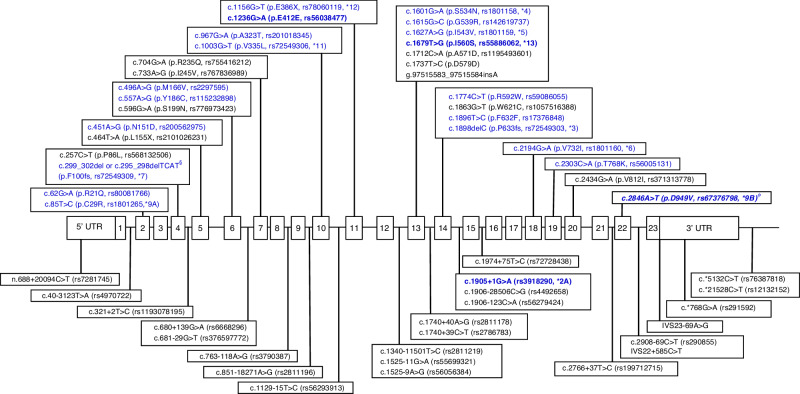
53 *DPYD* variants identified in our systematic review. Variants listed in the CPIC guideline are highlighted in blue. The four prominent European *DPYD* variants are in bold blue font. ^ɸ^c.2846A>T was not identified in our systematic review. ^§^c.299_302del is also known as c.295_298delTCAT (PharmGKB).

Across the 32 included studies, a total of 53 *DPYD* genetic variants were reported, of which 20 have been reported in the CPIC guideline [6] (Fig. 2). Genotype counts of variants reported in patients with severe fluoropyrimidine-related toxicity across the 5 ethnicities with details of all extracted data items are presented in Supplementary Table 5. Our in silico prediction results for all 53 *DPYD* variants identified are summarised in Table 3 with scores obtained from each in silico prediction tool detailed in Supplementary Table 6. In addition, 13 studies reported a combination of *DPYD* genetic variants at individual patient-level and we were able to identify 28 haplotype combinations as presented in Supplementary Table 7. Subsequent paragraphs in this section will focus on variants which were reported in more than 1 individual in each ethnicity with either: (1) CPIC-reported decreased or loss of DPD enzyme function or (2) unreported DPD enzyme function in the CPIC guideline but predicted to be deleterious by > 60% of the in silico tools we utilised. Variants which were excluded due to this filtering process and haplotype combinations are described in the Supplementary Results.

**Table 3:** In silico predictions and in vitro analyses of *DPYD* variants evaluated in our systematic review.

Chr:BP (GRCh38)	dbSNP rsID	HGVS^ɸ^ and star allele nomenclatures	Location, Molecular consequence	CPIC^a^		In silico predictions undertaken				Published in silico predictions		Published in vitro analyses^h^	
				Phenotype (LoE)	Dose recommendation (Classification)	Protein function/ structure^b^	Splicing^c^	Transcription factor binding^d^	miRNA binding^e^	DPYD-Varifier^f^	APF^f^	DPD enzyme activity	5-FU CL_int_
				Ref/Alt, Alt/Alt									
1:97921479	rs72981745	n.688+20094C>T	5’US			N/A	N/A	TF (1 of 3) NTF (2 of 3)	N/A				
1:97886497	rs4970722	c.40-3123T>A	Intron 1			N/A	SC (1 of 4) NSC (3 of 4)	N/A	N/A				
1:97883352	rs80081766	c.62G>A, p.Arg21Gln	Exon2, Missense	NM, NM (M)	No Change (S)	D (8 of 12) B (4 of 12)	SC (2 of 4) NSC (2 of 4)	N/A	N/A			NF [57]	
1:97883329	rs1801265	c.85T>C, p.Cys29 Arg, *9A	Exon2, Missense	NM, NM (H)	No Change (S)	D (1 of 10) B (9 of 10)	NSC (4 of 4)	N/A	N/A		Neutral	13% ↑ [56] 21% ↓ [58]	63.9% ↓ [59]
1:97740456	rs568132506	c.257C>T, p.Pro86Leu	Exon 4, Missense			D (12 of 12)	SC (2 of 4) NSC (2 of 4)	N/A	N/A			97% ↓ [42]	
1:97740411-97740418	rs72549309	c.299_302del or c.295_298delTCAT^§^, p.Phe100fs, *7	Exon 4, Frameshift	IM, PM (M)	50% ↓, Avoid (S, S)	D (3 of 3)	NSC (3 of 3)	N/A	N/A		Deleterious	LoF [57]	
1:97740390	rs1193078195	c.321+2T>C	Intron 4, Splice donor			N/A	SC (2 of 4) NSC (2 of 4)	N/A	N/A				
1:97721542	rs200562975	c.451A>G, p.Asn151Asp	Exon 5, Missense	NM, NM (W)	No change (S)	D (12 of 12)	NSC (4 of 4)	N/A	N/A		Deleterious	NF [57]	7% ↑ [45] 33% ↓ [59]
1:97721529	rs2101026231	c.464T>A, p.Leu155Ter	Exon 5, Nonsense			D (5 of 6) B (1 of 6)	SC (1 of 4) NSC (3 of 4)	N/A	N/A				
1:97699535	rs2297595	c.496A>G, p.Met166Val	Exon 6, Missense	NM, NM (M)	No change (S)	D (9 of 12) B (3 of 12)	SC (1 of 4) NSC (3 of 4)	N/A	N/A		Deleterious	20% ↑ [57] 23% ↓ [58]	22.7-38% ↓ [45, 59]
1:97699474	rs115232898	c.557A>G, p.Tyr186Cys	Exon 6, Missense	IM, IM (M)	50% ↓, 50% ↓ (M, S)	D (9 of 12) B (3 of 12)	SC (1 of 4) NSC (3 of 4)	N/A	N/A		Deleterious	15-29% ↓ [57, 65]	
1:97699435	rs776973423	c.596G>A, p.Ser199Asn	Exon 6, Missense			D (11 of 12) B (1 of 12)	SC (1 of 4) NSC (3 of 4)	N/A	N/A	Deleterious			
1:97699212	rs6668296	c.680+139G>A	Intron 6			N/A	NSC (4 of 4)	N/A	N/A				
1:97691827	rs376597772	c.681-29G>T	Intron 6			N/A	NSC (4 of 4)	N/A	N/A				
1:97691775	rs755416212	c.704G>A, p.Arg235Gln	Exon 7, Missense			D (12 of 12)	SC (1 of 4) NSC (3 of 4)	N/A	N/A	Deleterious			
1:97691746	rs767836989	c.733A>G, p.Ile245Val	Exon 7, Missense			D (1 of 12) B (11 of 12)	SC (1 of 4) NSC (3 of 4)	N/A	N/A	Neutral			
1:97679300	rs3790387	c.763-118A>G	Intron 8			N/A	SC (1 of 4) NSC (3 of 4)	N/A	N/A				
1:97613437	rs2811196	c.851-18271A>G	Intron 9			N/A	NSC (4 of 4)	N/A	N/A				
1:97593379	rs201018345	c.967G>A, p.Ala323Thr	Exon 10, Missense	NM, NM (W)	No change (S)	D (3 of 12) B (9 of 12)	SC (1 of 4) NSC (3 of 4)	N/A	N/A			NF [57]	
1:97593343	rs72549306	c.1003G>T, p.Val335Leu, *11	Exon 10, Missense	NM, NM (M)	No change (S)	D (11 of 12) B (1 of 12)	SC (1 of 4) NSC (3 of 4)	N/A	N/A			NF [57]	
1:97573985	rs56293913	c.1129-15T>C	Intron 10			N/A	NSC (4 of 4)	N/A	N/A				
1:97573943	rs78060119	c.1156G>T, p.Glu386Ter, *12	Exon 11, Nonsense	IM, PM (M)	50% ↓, Avoid (S, S)	D (6 of 6)	SC (1 of 4) NSC (3 of 4)	N/A	N/A			LoF [57]	
1:97573863	rs56038477	c.1236G>A/HapB3, p.Glu412Glu	Exon 11, Synonymous	IM, IM (H)	50% ↓, 50% ↓ (M, S)	D (1 of 5) B (4 of 5)	NSC (4 of 4)	N/A	N/A				
1:97561245	rs2811219	c.1340-11501T>C	Intron 12			N/A	SC (1 of 4) NSC (3 of 4)	N/A	N/A				
1:97515952	rs55699321	c.1525-11G>A	Intron 12			N/A	NSC (4 of 4)	N/A	N/A				
1:97515950	rs56056384	c.1525-9A>G	Intron 12			N/A	NSC (4 of 4)	N/A	N/A				
1:97515865	rs1801158	c.1601G>A, p.Ser534Asn, *4	Exon 13, Missense	NM, NM (M)	No change (S)	D (8 of 11) B (3 of 11)	NSC (4 of 4)	N/A	N/A		Deleterious (False positive)^g^	36% ↑ [56] 21% ↓ [58]	
1:97515851	rs142619737	c.1615G>C, p.Gly539Arg	Exon 13, Missense	NM, NM (W)	No change (S)	D (11 of 12) B (1 of 12)	NSC (4 of 4)	N/A	N/A			NF [57]	
1:97515839	rs1801159	c.1627A>G, p.Ile543Val, *5	Exon 13, Missense	NM, NM (H)	No change (S)	D (1 of 12) B (11 of 12)	SC (1 of 4) NSC (3 of 4)	N/A	N/A		Neutral	NF [56, 58]	1.8% ↑ [45] 25.6% ↓ [59]
1:97515787	rs55886062	c.1679T>G, p.Ile560Ser, *13	Exon 13, Missense	IM, PM (M)	50% ↓, Avoid (S, S)	D (11 of 12) B (1 of 12)	NSC (4 of 4)	N/A	N/A		Deleterious	75% ↓ [56]	9.7% ↑ [59]
1:97515754	rs1195493601	c.1712C>A, p.Ala571Asp	Exon 13, Missense			D (9 of 11) B (2 of 11)	NSC (4 of 4)	N/A	N/A				
1:97515729		c.1737T>C, p.Asp579Asp	Exon 13, Synonymous			B (4 of 4)	NSC (2 of 2)	N/A	N/A				
1:97515583		g.97515583_97515584insA	Intron 13, Insertion			N/A	Insufficient reported information for prediction	N/A	N/A				
1:97515686	rs2811178	c.1740+40A>G	Intron 13			N/A	NSC (4 of 4)	N/A	N/A				
1:97515687	rs2786783	c.1740+39C>T	Intron 13			N/A	NSC (4 of 4)	N/A	N/A				
1:97450190	rs59086055	c.1774C>T, p.Arg592Trp	Exon 14, Missense	IM, PM (W)	50% ↓, Avoid (S, S)	D (12 of 12)	SC (1 of 4) NSC (3 of 4)	N/A	N/A		Deleterious	>90% ↓ [57]	95-98% ↓ [45, 59]
45,591:97450101	rs1057516388	c.1863G>T, p.Trp621Cys	Exon 14, Missense			D (12 of 12)	SC (1 of 4) NSC (3 of 4)	N/A	N/A				
1:97450068	rs17376848	c.1896T>C, p.Phe632Phe	Exon 14, Synonymous	NM, NM (M)	No change (S)	D (1 of 5) B (4 of 5)	NSC (4 of 4)	N/A	N/A				
1:97450066-97450067	rs72549303	c.1898delC, p.Pro633fs, *3	Exon 14, Frameshift	IM, PM (M)	50% ↓, Avoid (S, S)	D (3 of 3)	NSC (3 of 3)	N/A	N/A		Deleterious	LoF [57]	
1:97450058	rs3918290	c.1905+1G>A, *2 A	Intron 14, Exon 14 skipping, Splice donor	IM, PM (H)	50% ↓, Avoid (S, S)	D (2 of 2)	SC (2 of 4) NSC (2 of 4)	N/A	N/A			LoF [56]	
1:97410967	rs4492658	c.1906-28506C>G	Intron 14			N/A	SC (1 of 4) NSC (3 of 4)	N/A	N/A				
1:97382584	rs56279424	c.1906-123C>A	Intron 14			N/A	NSC (4 of 4)	N/A	N/A				
1:97382318	rs72728438	c.1974+75T>C	Intron 15			N/A	SC (1 of 4) NSC (3 of 4)	N/A	N/A				
1:97305364	rs1801160	c.2194G>A, p.Val732Ile, *6	Exon 18, Missense	NM, NM (M)	No change (S)	D (6 of 11) B (5 of 11)	NSC (4 of 4)	N/A	N/A		Neutral	NF [56, 57] 30% ↓ [58]	14-21% ↑ [45, 59]
1:97234991	rs56005131	c.2303C>A, p.Thr768Lys	Exon 19, Missense	NM, NM (W)	No change (S)	D (7 of 12) B (5 of 12)	NSC (4 of 4)	N/A	N/A		Deleterious	NF [57]	52-56% ↓ [45, 59]
1:97234860	rs371313778	c.2434G>A, p.Val812Ile	Exon 20, Missense			D (4 of 12) B (8 of 12)	SC (1 of 4) NSC (3 of 4)	N/A	N/A				
1:97098452	rs199712715	c.2766+37T>C	Intron 22			N/A	NSC (4 of 4)	N/A	N/A				
1:97079215	rs290855	c.2908-69A>G	Intron 22			N/A	NSC (4 of 4)	N/A	N/A				
		IVS22 + 585C>T	Intron 22			N/A	Insufficient reported information for prediction	N/A	N/A				
		IVS23-69A>G	Intron 23			N/A	Insufficient reported information for prediction	N/A	N/A				
1:97078208	rs291592	c.*768G>A	3’ UTR			N/A	NSC (2 of 2)	N/A	miR (2 of 2)				
1:97073844	rs76387818	c.*5132C>T	~4 kb 3’of *DPYD*			N/A	SC (1 of 3) NSC (2 of 3)^k^	N/A	N/A				
1:97057448	rs12132152	c.*21528C>T	~20 kb 3’of *DPYD*			N/A	SC (1 of 3) NSC (2 of 3)^k^	N/A	N/A				

*Alt/Alt* Homozygous variant carrier, *APF* ADME-optimised Prediction Framework, *B* Benign, *BP* Base pair position, *Chr* Chromosome, *CL*_*int*_ Intrinsic Clearance, *CPIC* Clinical Pharmacogenetics Implementation Consortium, *C/P* cannot predict, *D* Deleterious, *DPYD* Dihydropyrimidine dehydrogenase gene, *H* High, *HGVS* Human Genome Variation Society, *I* Increased, *IM* Intermediate Metabolizer, *LoE* Levels of evidence, *LoF* Loss of function, *M* Moderate, *miR* miRNA binding site, *N* Normal/Neutral, *N/A* Not applicable, *NF* Normal Function, *NM* Normal Metabolizer, *NSC* No change in splicing, *NTF* No change in transcription factor binding, *PM* Poor Metabolizer, *R* Reduced, *Ref/Alt* Heterozygous variant carrier, *S* Strong, *SC* Change in splicing, *TF* Change in transcription binding, *US* Upstream, *UTR* Untranslated region, *W* Weak.

^ɸ^Reference sequences NM_000110.4 and NP_000101.2 were used.

^§^c.299_302del is also known as c.295_298delTCAT (PharmGKB).

^a^In accordance with the CPIC guideline for fluoropyrimidines and *DPYD*, the likely DPD phenotype based on *DPYD* genotype, the grading levels of evidence (LoE) linking genotype to phenotype, the fluoropyrimidine dose recommendations based on genotype/phenotype, and the classification of fluoropyrimidine dose recommendations are reported.

^b^Effect on DPD protein function or structure was predicted by Sorting Intolerant From Tolerant (SIFT), Polymorphism Phenotyping v2 (PolyPhen-2), MutPred2, Mendelian Clinically Applicable Pathogenicity (M-CAP), Cancer-Related Analysis of Variants Tool (CRAVAT), Rare Exome Variant Ensemble Learner (REVEL), MutationAssessor, MetaLR, Functional Analysis Through Hidden Markov Models (FATHMM), MutationTaster2021, Combined Annotation Dependent Depletion (CADD) and PredictSNP2. In silico prediction scores were classified as deleterious (D) if variant was predicted to be deleterious, damaging, probably damaging, possibly damaging, pathogenic, possibly pathogenic, likely disease-causing, high deleterious probability, or medium deleterious probability; and benign (B) if variant was predicted to be benign, tolerated, likely benign, low deleterious probability, or neutral.

^c^Effect on splicing was predicted using SpliceAI, Human Splicing Finder (HSF), NNSplice, and SpliceRover. In silico prediction results were summarised as follows: SC = Change in splicing; NSC = No change in splicing.

^d^Effect on transcription factor binding was predicted using PROMO, SNP2TFBS and sTRAP. *In siilico* prediction results were summarised as follows: TF = change in transcription factor binding; NTF = No change in transcription factor binding.

^e^Effect on binding affinity for target miRNAs was predicted using the PolymiRTS database and MicroSNiPer. In silico prediction results were summarised as follows: miR = miRNA binding site created.

^f^Published data from previously developed in silico functional prediction models, DPYD-Varifier [53] and the ADME-optimised Prediction Framework (APF) [54, 55], were extracted.

^g^Identified as false positive by authors of APF [55].

^h^Published functional data on DPD enzyme activity and 5-FU reduction from in vitro experiments transiently expressed with *DPYD* variants using HEK293T/c17 cells and substrate 5-FU [42, 56, 57], HEK293T Flp-In cells and substrate thymine [58], and 293FT cells and substrate 5-FU [45, 59] were reported. The scores were assigned as follows: LoF = Loss of function; ↓ = Reduced; ↑ = Increased; NF = Normal function.

^k^In silico prediction was performed using *DPYD* intron 22 variant rs142861208 which is in perfect LD (*r*^2^ = 1) with identified variant.

#### African American

19 *DPYD* variants (2 missense, 2 frameshift, 11 intronic, one 5′-upstream, one 3’UTR, two 3′-downstream) were reported across 3 case studies [60, 62, 63] and 1 cohort study [64] conducted in patients of African American ancestry in the United States (Supplementary Table 5).

Heterozygous carriage of the missense variant c.557A>G (Tyr186Cys) was reported in all 3 case studies [60, 62, 63]. This variant has a mean prevalence of ~2% in reference populations of African descent (Supplementary Table 1) [40, 41] and the presence of either 1 or 2 copies of the c.557A>G variant allele is considered to cause a decrease in DPD enzyme function (intermediate metaboliser) by the CPIC guideline with moderate strength of evidence. Up to 75% of the in silico prediction tools we utilised predicted this variant to be deleterious and this variant was classified as deleterious by APF (Table 3, Supplementary Table 6). In vitro functional analysis containing the Tyr186Cys amino acid substitution showed between ~15% to 29% reduction in DPD enzyme activity relative to the wild-type (Table 3, Supplementary Table 6) [57, 65]. In addition, in a healthy cohort of African Americans, DPD enzyme activity in PBMCs was found to be 46% lower in heterozygous carriers compared to non-variant carriers [66]. Maharjan and colleagues (2019) did not include c.557A>G genetic testing in their cohort of African American patients [64].

#### East Asian

A total of 30 *DPYD* variants (2 nonsense, 15 missense, 3 synonymous, 2 splice donor, and 8 intronic) were reported in patients of East Asian ancestry which included 5 cohort studies [67–71] and 2 case reports from China [72, 73], 2 cohort studies [46, 48] and 3 case reports from Japan [61, 74, 75], 1 cohort study from Korea [76], and 1 cohort study from Thailand [77] (Supplementary Table 5).

Amongst the 30 variants identified, 15 have been reported in the CPIC guideline including 3 loss of function variants, c.1156G>T (Glu386Ter), c.1774C>T (Arg592Trp) and c.1905+1G>A, with moderate, weak, and high strength of evidence respectively. Heterozygous carriers of 1 of these 3 variants lead to decreased enzyme function and are classified as intermediate metabolisers by CPIC; while homozygous carriers of either of these 3 variants lead to loss of enzyme function and are classified by CPIC as poor metabolisers. In reference populations of East Asian descent, these 3 variants are rare with zero MAF observed for c.1156G>T and c.1905+1G>A, and a MAF of 0.1% for c.1774C>T (Supplementary Table 1) [40, 41].

Heterozygous carriage of the truncating c.1156G>T variant was reported in three Japanese patients, two from case reports who both exhibited >10 fold decrease in PBMC DPD enzyme activity in comparison to normal/healthy individuals [74, 75], and one from a cohort study where heterozygous carriage of 1 of the 7 rare pathogenic *DPYD* variants, c.596G>A, c.733A>G, c.914C>A, c.1156G>T, c.1666A>C, c.1712C>A, or c.1863G>T was significantly associated with grade 3–4 toxicity in comparison to patients without the 7 rare variants (OR = unreported; *p* = 0.0271; Supplementary Table 5) [48]. 100% of the in silico prediction tools we utilised predicted c.1156G>T to be deleterious and published in vitro expression analysis reported complete loss of DPD enzyme activity (Table 3, Supplementary Table 6) [57, 78].

Two patients, one from a Korean cohort study and one from a Thai cohort study, were heterozygous for the nonsynonymous variant c.1774C>T [76, 77]. 100% of the in silico prediction tools we utilised predicted c.1774C>T to be deleterious and the APF classified this variant as deleterious (Table 3, Supplementary Table 6). Previously published in vitro functional characterisation of c.1774C>T reported >90% reduction in DPD catalytic activity compared to the wild-type (Table 3, Supplementary Table 6) [45, 57, 59, 78].

Heterozygous carriers of the intron 14 splice donor variant c.1905+1G>A were reported in one Thai cohort patient [77] and 14 Chinese cohort patients in which significantly higher incidences of grade 3–4 myelosuppression, hand-foot syndrome, diarrhoea, gastrointestinal reactions and mucositis were observed (OR = unreported; *p* < 0.001 for each severe side effect) compared to wild-type carriers [70]. 100% of the in silico prediction tools we utilised predicted this variant to be deleterious and published in vitro expression analysis reported c.1905+1G>A to be catalytically inactive (Table 3, Supplementary Table 6) [56, 78].

Two Chinese patients from a cohort study, one with grade 4 bone marrow inhibition (BMI) and one with grade 4 BMI and grade 4 gastrointestinal toxicity, were reported to be heterozygous carriers for the nonsense variant, c.464T>A (Leu155Ter). This variant is not reported in the CPIC guideline. The DPD enzyme activity in PBMCs from both patients was ~45% lower than that in non-carriers with Grade 1–2 toxicity (Supplementary Table 5) [71]. In addition, when c.464T>A was analysed in composite with c.85T>C and c.2194G>A, the carriage of either c.464T>A, c.85T>C, and/or c.2194G>A was associated with an increased incidence of bone marrow toxicity (OR = 24; *p* = 0.0001) and gastrointestinal toxicity (OR = 8; *p* = 0.0019) in comparison to non-variant carriers (Supplementary Table 5) [71]. Over 80% of the in silico prediction tools we used predicted the c.464T>A to be deleterious (Table 3, Supplementary Table 6). No allele frequency information in reference populations of East Asian descent and other ancestries has been reported for this variant (Supplementary Table 1) [40, 41].

#### Latin American

Only 1 cohort study from Chile was identified in the Latin American population [79] and the authors detected 3 missense *DPYD* polymorphisms considered to have normal DPD enzyme function by the CPIC guideline, c.85T>C, c.496A>G, and c.1627A>G (Supplementary Table 5, Supplementary Results).

#### Middle Eastern

13 *DPYD* variants (1 splice donor, 8 missense, 4 intronic) were reported in patients of Middle Eastern ancestry. There were 2 cohort studies from Tunisia [80, 81], 1 cohort study from Jordan [82], 1 case report from Lebanon [83], and 1 case series from Saudi Arabia [84] (Supplementary Table 5). None of the variants passed our filtering process (Supplementary Results).

#### South Asian

7 *DPYD* variants (6 missense, 1 splice donor) were reported in patients of South Asian ancestry across 5 cohort studies from India [85–89], one Indian case series [90], one case study of an Indian patient in the USA [91], and 1 cohort study from Bangladesh [92] (Supplementary Table 5).

With a prevalence of 0.3–1.5% in reference populations of South Asian descent (Supplementary Table 1) [40, 41], the splice donor variant c.1905+1G>A was reported in patients from Bangladesh and India [85, 86, 88–90, 92]. The Bangladeshi cohort study reported a significant association with anaemia (OR = 4.7, *p* = 0.042), neutropenia (OR = 6.47, *p* = 0.018), thrombocytopaenia (OR = 8.08, *p* = 0.05), nausea (OR = 10.06, *p* = 0.012), and diarrhoea (OR = 5.76, *p* = 0.026) when patients with grade 3–4 toxicities were compared to patients with grade ≤2 toxicities [92]. The Bangladeshi cohort study genotyped for only the c.1905+1G>A variant, and the occurrence of other mutations was not investigated. One of the four Indian cohort studies reported a decreased incidence of mucositis (*p* = 0.016) and diarrhoea (*p* = 0.006) in *DPYD* variant carriers of either c.85 T>C, c.496A>G, c.1627A>G, c.1905+1G>A and/or c.2194G>A after 50% capecitabine dose reduction in cycle 2 of chemotherapy [88].

## Discussion

This systematic review has identified numerous variants in the *DPYD* gene which have been reported in non-European individuals with severe and sometimes fatal toxicity associated with the use of fluoropyrimidines. In the UK and EU, testing for 4 *DPYD* genetic variants is undertaken before the use of fluoropyrimidines [36, 37] — in England, we currently do 38,000 tests per year. This is an important success story for the implementation of pharmacogenomics, but there is still a need to improve the testing pathway, both in terms of increasing the number of genetic variants tested, and ensuring that we are not disadvantaging particular ethnic groups.

It is interesting to note that our systematic review has identified 3 of the 4 *DPYD* variants tested in the UK and EU [36, 37], in non-European individuals. The c.1905+1G>A variant, which leads to exon 14 skipping, has been reported in 1 Thai [77], 14 Chinese [70], 1 Lebanese [83], 7 Bangladeshi [92] and 18 Indian [85–90] patients with fluoropyrimidine-related toxicity. The frequency of this variant is 0% in East Asian reference populations, 0.3% in Middle Eastern reference populations, and 0.3–1.5% in South Asian reference populations [40, 41]. The c.1679T>G and c.1236G>A/HapB3 variants have been reported in 1 Tunisian patient [81] and 1 Thai patient [77], respectively. The prevalence of c.1679T>G is 0% in Middle Eastern reference populations [41] and the frequency of c.1236G>A/HapB3 ranges from 0.01-0.1% in East Asian reference populations [41]. According to the 2021 UK census [93], South Asians, East Asians, and Arabs represent 6.7%, 1.3%, and 0.6% of the UK population, respectively, and thus they will benefit from the genetic testing which is offered to all patients in the UK if they require treatment with 5-FU or its analogues.

Clearly, there are other variants in these ethnic groups which need further investigation. For example, in South Asians and Middle Easterners, our systematic review identified single occurrence of missense variants c.704G>A (p.Arg235Gln, rs755416212) [91] and c.257C>T (p.Pro86Leu, rs568132506) [84], respectively. These variants are not reported in the CPIC guideline but are predicted to be deleterious by 100% of the in silico tools we used, with one research study reporting significant reduction of DPD activity in vitro (97% decrease) with the c.257C>T variant [42]. Further functional work and greater interrogation of patients who have had toxicity is warranted to confirm these findings and to identify other functionally relevant variants.

Our systematic review has identified 3 case studies detecting the c.557A>G variant (rs115232898, p.Tyr186Cys) in African Americans with severe 5-FU-related toxicity [60, 62, 63], one of which was potentially fatal [60]. In addition, in an editorial which was not eligible for inclusion in our systematic review, this variant was reported in an African-Caribbean patient with severe 5-FU-related toxicity [94]. This is a nonsynonymous variant located on exon 6 where in vivo [66] and in vitro studies [57, 65] have shown between ~15% to 46% reduction in DPD activity relative to wildtype. The CPIC guideline recommends 50% reduction in fluoropyrimidine starting dose for heterozygous or homozygous carriers of the c.557A>G variant allele with moderate and strong classification, respectively. Data from the 1000 Genomes Project Phase 3 confirms that c.557A>G is mainly found in African populations (Afro-Caribbeans in Barbados, African Americans in southwest United States, Yoruba in Ibadan (Nigeria), Luhya in Webuye (Kenya), Gambian in Western Divisions in the Gambia, Mende in Sierra Leone, and Esan in Nigeria), with allele frequency ranging between 1–4% [40]. This variant is virtually non-existent in Europeans, East Asians and South Asians. In the United States, the Mayo Clinic and several commercial laboratories includes c.557A>G in their pre-treatment *DPYD* testing to identify individuals at increased risk of toxicity when considering fluoropyrimidine chemotherapy treatment. However, this variant is currently not included in the UK NHS *DPYD* genetic testing. In the 2021 UK Census, 4% (2.4 million) of the total population in England and Wales identified their ethnic group within the “Black, Black British, Black Welsh, Caribbean or African” category [93].

Our systematic review also shows that few novel variants in the *DPYD* gene have been reported in Middle Eastern [82] populations with a paucity of data in Latin American populations [79], highlighting the need for more studies in these populations. Indeed, further studies are needed in all populations (European and non-European) to fully understand the spectrum of harmful mutations which occur in this gene. This will require careful identification and assessment of patients with toxicity caused by 5-FU or its analogues, and subsequent sequencing of the *DPYD* gene together with functional characterisation of any mutations identified. To this end, we have initiated a programme of work (called “DPYD-International”) which has the aim to identify affected patients globally so that evidence can be generated to optimise the pathway for *DPYD* genetic screening to maximise benefits for all populations and minimise any unintended inequalities.

Previous studies have shown that *DPYD* intermediate and poor metabolizers receiving conventional doses of fluoropyrimidine are at significantly higher risk for severe toxicity and treatment-related mortality [31, 32] and pre-treatment testing followed by genotype-guided dose reduction in variant carriers significantly reduces toxicity and mortality risks [31–35], and associated hospitalisations [32, 95–97]. This strategy has also been shown to be cost-effective. For example, a UK-based study of an extended *DPYD* genetic panel showed that genotyping was dominant over standard of care, with a saving of £78,000 per patient over a lifetime [98]. Two other studies, one from Canada [99] and another from Iran [100], have also shown pre-prescription *DPYD* genotyping to be cost saving, while studies from the US [95] and Spain [101] showed it to be cost-effective.

Our systematic review has limitations. The proportion of non-English language publications varied across the four electronic databases we utilised: Embase (OVID) — 0.9%, Web of Science — 3.2%, MEDLINE (PubMed) — 5%, Scopus — 6.6%. We had to rely on a mixture of different study types, including case series, case reports and cohort studies, to identify affected patients. Clearly this represents selective reporting, and many patients with important variants are either not reported, or more likely not genotyped or sequenced due to variability in genetic testing methods and target gene regions/variants. This may be particularly the case with fatal cases where DNA may not be available for retrospective testing. It is therefore important future studies are designed to identify and sequence these patients to evaluate the full spectrum of mutations associated with toxicity from 5-FU or its analogues. An individual patient-level analysis might have been more rewarding but the number of studies conducted in each ethnicity was small and some authors did not respond to invitations to provide data clarification. Although large-scale biomedical databases such as the UK Biobank has been designed to facilitate health-related research, secondary care data relating to severe fluoropyrimidine-related toxicity are not available in these databases. For many of the variants identified in this review, the functional consequences are unknown; very few studies have measured in vivo DPD activity and furthermore, different methods for measuring DPD activity were used. We have undertaken a comprehensive in silico evaluation of the likely functional consequences of the mutations, but further functional evaluation will be needed for many of the variants. Notably, our systematic review has identified a number of patients carrying more than one *DPYD* variant and in particular one African American carrying 2 loss-of-function variants c.299_302del/c.295_298delTCAT and c.1898delC in addition to the decreased function variant c.557A>G (Supplementary Results) [63]; how the co-expression of functional *DPYD* variants affects overall DPD activity and the consequences for the severity of fluoropyrimidine-related toxicity remains to be elucidated. Our focus has been on the *DPYD* gene, but there are other potential genes (e.g. MIR27A, TYMS, ENOSF1, MHTFR) which may be important in predisposing to toxicity from the fluoropyrimidines, and these will need a separate evaluation.

In conclusion, our systematic review has focused on non-European patients and has identified numerous variants in the *DPYD* gene which have been reported in patients with severe toxicity after treatment with 5-FU or its oral analogues. The UK is an increasingly multi-cultural and ethnically diverse society with 18% of the population from non-European ethnic groups but we test for 4 variants which have been identified from studies undertaken in European populations. However, our analysis shows that 3 of these 4 variants are also important in South Asian, East Asian and Middle Eastern individuals. From the evidence gathered, and based on practice elsewhere in the world, we feel that it would be important to extend *DPYD* genetic testing in the UK NHS to include the c.557A>G variant which has been identified in individuals of African ancestry. The other variants described in this systematic review need further evaluation for incorporation into the testing pathways either in the UK or elsewhere including other multi-ethnic countries like the EU, USA and Canada, where non-Europeans represent 10–15%, 24.5%, 10.8% of the population, respectively. If sequencing becomes the standard method for characterising *DPYD* variation, we hope the information contained within this systematic review will be of use to diagnostic labs and policy makers.

### Supplementary information

Supplementary-Methods-Results-Discussion

Supplementary Tables

PRISMA 2020 Checklist

## Data Availability

Data used in this review is provided in Supplementary Appendices; any additional data are available upon request to the corresponding author.
