# Supplementary material for: DPYD genetic polymorphisms in non-European patients with severe fluoropyrimidine-related toxicity: a systematic review
Source: Br J Cancer. 2024 Jun 17;131(3):498–514. doi: 10.1038/s41416-024-02754-z (PMC11300675; doi:10.1038/s41416-024-02754-z)
Supplement: Supplementary file 1 — Supplementary-Methods-Results-Discussion [file 41416_2024_2754_MOESM1_ESM.docx]

**Supplementary Methods**

**Quality assessment**

For clinical research studies, the quality of research design, quality and validity of research findings, and appropriateness of statistical analysis undertaken were evaluated. For case series and case reports, reporting of demographics, clinical diagnosis, fluoropyrimidine chemotherapy treatment, clinical symptoms including severe toxicity presentation, intervention/treatment procedures, post-intervention clinical condition, and genetic results were assessed.

**Data extraction**

Data items collected included study design, study location, demographics of the patient population including age, gender and ethnicity, cancer type, fluoropyrimidine agent (5-FU, capecitabine or tegafur), chemotherapy regimen (fluoropyrimidine monotherapy or combination chemotherapy), *DPYD* genetic testing method, *DPYD* variants evaluated, number of patients with severe fluoropyrimidine-related toxicity evaluated for each *DPYD* variant, details of severe toxicity manifestations, severe toxicity grading tool, grade of severe toxicity, the statistical association of *DPYD* variant with severe fluoropyrimidine-related toxicity, DPD phenotyping method, DPD enzyme activity level, and details of fluoropyrimidine dose reduction and/or discontinuation after the development of severe fluoropyrimidine-related toxicity. We noticed that in some studies, a combination of *DPYD* genetic variants were reported at individual patient-level and we were able to identify *DPYD* haplotypes (although in depth assessment of LD was not possible as raw genotypic data were not obtained). As such, the various potential/partial *DPYD* haplotype combinations and the corresponding number of patients with severe fluoropyrimidine-related toxicity identified for each *DPYD* haplotype were extracted.

***In silico* prediction**

The impact of exonic *DPYD* variants on DPD protein function or structure was predicted using SIFT (Sorting Intolerant From Tolerant) ^1,2^, PolyPhen-2 (Polymorphism Phenotyping v2) ^3,4^, MutPred2 ^5,6^, M-CAP (Mendelian Clinically Applicable Pathogenicity) ^7^, MutationTaster2021 ^8^, CADD (Combined Annotation Dependent Depletion) ^9-11^, CRAVAT (Cancer-Related Analysis of Variants Toolkit) ^12-15^, REVEL (Rare Exome Variant Ensemble Learner) ^16^, MutationAssessor ^17,18^, MetaLR ^19,20^, FATHMM (Functional Analysis Through Hidden Markov Models) ^21-23^, and PredictSNP2 ^24^.

The effect of *DPYD* variants on splicing was predicted using SpliceAI ^25^, Human Splicing Finder (HSF) ^26^, NNSplice ^27^, and SpliceRover ^28^.

To predict if variants located in the 5’ upstream (5’ US) region affect transcription factor binding, PROMO ^29,30^, SNP2TFBS ^31^ and sTRAP ^32^ were used. To test whether variants located in 3’ untranslated region (3’ UTR) can change the binding affinity for target miRNAs, the PolymiRTS database ^33,34^ and MicroSNiPer tool ^35^ were used.

**Published *in silico* functional predictions**

The DPYD-Varifier was developed using both machine learning and *in vitro* functional data on 156 missense *DPYD* variants to create a model with 85% accuracy ^36^. The ADME-optimised Prediction Framework (APF) can quantitatively predict the functional impact of loss-of-function and functionally neutral variants with 93% sensitivity and specificity ^37,38^.

**Supplementary Results**

***DPYD* genetic variants, haplotypes and *in silico* predictions**

***African American***

Of the 19 *DPYD* variants reported, single occurrence was observed with 17 variants ^39,40^, and 15 variants are currently unreported in the CPIC guideline (Supplementary Table 5).

*CPIC-reported loss of DPD enzyme function*

Two rare frameshift variants considered by CPIC to lead to complete loss of DPD enzyme function with moderate level of evidence, c.299_302del/c.295_298delTCAT (Phe100fs) in exon 4 and c.1898delC (Pro633fs) in exon 14, were present together with c.85T>C, c.557A>G, four intronic and two 3’downstream *DPYD* variants in one case study patient ^40^ (Supplementary Table 5, Supplementary Table 7). The patient was reported to be homozygous for the c.299_302del/c.295_298delTCAT variant allele and heterozygous for c.1898delC. The patient developed severe pancytopenia following first cycle of mFOLFOX6 but subsequent cycles with no 5-FU bolus and 50% dose reduction in 5-FU infusion was well tolerated. The MAF of each of these 2 rare frameshift variants is 0.001% and 0% in the latest release of gnomAD (v4.0.0) African reference population (Supplementary Table 1) ^41^. 100% of the *in silico* tools we utilised predicted both c.299_302del/c.295_298delTCAT and c.1898delC to be deleterious and APF classified both variants as deleterious (Table 3, Supplementary Table 6). In addition, both variants have been previously reported to lead to complete loss of DPD function using the HEK293/c17 *in vitro* expression system (Supplementary Table 6) ^42^.

*CPIC-reported normal DPD enzyme function*

With a MAF of 40-44% in reference populations of African descent (Supplementary Table 1) ^41,43^, the normal function missense variant c.85T>C (Cys29Arg) was reported in 2 case studies ^39,40^ and in ~11 African American patients in a cohort study ^44^ (Supplementary Table 5).

*Unreported in CPIC*

None of the 11 intronic variants were predicted to affect splicing by > 75% of the *in silico* splicing prediction tools we utilised (Table 3, Supplementary Table 6). The two 3’-downstream variants are located >4 kb downstream of *DPYD*. They are both in perfect linkage disequilibrium (LD; r^2^ = 1) with a *DPYD* intron 22 variant, rs142861208, which was utilised in the *in silico* prediction analysis and was not predicted to affect splicing by >65% of the splicing prediction tools used (Table 3, Supplementary Table 6).

Among the 3 transcription factor binding prediction tools used, only the PROMO program predicted the 5’-upstream variant, n.688+20094C>T (rs72981745), to affect transcription factor binding and no significant difference in transcription factor binding was predicted by SNP2TFBS and sTRAP (Table 3, Supplementary Table 6).

The 3’UTR variant, c.*768G>A (rs291592), was predicted to affect miRNA binding (Table 3, Supplementary Table 6).

*Haplotypes*

Two haplotype combinations were identified in 2 case studies respectively ^39,40^. Both haplotypes contain the decreased function variant c.557A>G and the normal function missense variant c.85T>C (Cys29Arg) (Supplementary Table 7).

***East Asian***

Of the 15 *DPYD* variants with CPIC-reported DPD enzyme function, 12 are considered normal function variants (Supplementary Table 5).

*CPIC-reported normal DPD enzyme function*

Two common variants, the missense c.1627A>G (Ile543Val) and synonymous c.1896T>C (Phe632Phe) with respective MAF of 25-27% and 11-13% in reference populations of East Asian descent (Supplementary Table 1) ^43,45^ were reported in patients from all 4 East Asian countries included in our systematic review – China, Japan, Korea, and Thailand (Supplementary Table 5). Of the 8 cohort studies which genotyped/sequenced c.1627A>G ^46-53^, two Chinese studies found significant association with increased incidences of grade 3-4 toxicity compared to wild-type carriers ^49,52^ and the Thai cohort study reported significant difference in actual absolute neutrophil count change (*p* = 0.011) and percentage of neutrophil change (*p* = 0.009) in homozygous variant carriers compared to wild-type ^51^; but no significant association was observed in the other 5 cohort studies ^46-48,50,53^. Among the 3 cohort studies which sequenced c.1896T>C, no significant association was reported ^46,49,51^.

With a prevalence of 5-9% in reference populations of East Asian descent (Supplementary Table 1) ^43,45^, the missense polymorphism c.85T>C (Cys29Arg) was identified in Chinese, Japanese and Korean patients (Supplementary Table 5). Although c.85T>C was genotyped in the Thai cohort, none of the patients carried the variant ^51^. Mixed association findings were reported for c.85T>C – three Chinese cohort studies found significant increased risk of severe fluoropyrimidine-related toxicity in c.85T>C variant carriers compared to wild-type carriers ^50,52,54^ while 4 other cohort studies did not find any significant association ^46-48,53^.

Carriers of the nonsynonymous variant c.496A>G (Met166Val) were reported in 2 Japanese cohort studies ^48,53^ and 1 Korean cohort study ^46^ but no significant association with severe toxicity was observed. Of note, c.496A>G was not genotyped in the Thai cohort and as such, the carriage status of c.496A>G cannot be determined ^51^. The MAF of this variant in reference populations of East Asian descent is ~2% (Supplementary Table 1) ^43,45^.

The missense variant c.2194G>A (Val732Ile) has a MAF of 1.5-1.9% in reference populations of East Asian descent (Supplementary Table 1) ^43,45^. Heterozygous carriers of this variant were identified in one Chinese cohort study and two Japanese cohort studies. No significant association was found in either of the Japanese cohorts ^48,53^. However, the Chinese cohort study found significantly higher incidence of severe bone marrow inhibition (OR = 24, *p* = 0.0001) and gastrointestinal toxicity (OR = 8, *p* = 0.0019) in carriers of either c.85T>C, c.464T>A, and/or c.2194G>A compared to wild-type ^54^. In addition, the DPD enzyme activity in PBMCs from the 3 Chinese heterozygous c.2194G>A patients was ~38% lower than that in non-carriers with Grade 1-2 toxicity (Supplementary Table 5) ^54^.

The remaining 7 CPIC-reported normal function variants are rare and were reported in isolated cases. The missense variant c.967G>A (Ala323Thr) and synonymous variant c.1236G>A/HapB3 (Glu412Glu) were reported in two Thai patients, respectively ^51^. Five other missense variants, c.62G>A (Arg21Gln), c.451A>G (Asn151Asp), c.1003G>T (Val335Leu), c.1615G>C (Gly539Arg) and c.2303C>A (Thr768Lys) were reported in Japanese patients from 2 case reports and 2 cohort studies ^48,53,55,56^. These 7 rare variants have MAF < 1.5% in reference populations of East Asian descent (Supplementary Table 1) ^43,45^.

*Unreported in CPIC*

Among the 15 *DPYD* variants which are absent in the current CPIC guideline, 14 did not pass our filtering process which include one nonsense variant c.464T>A (Leu155Ter), four non-synonymous variants, c.596G>A (Ser199Asn), c.733A>G (Ile245Val), c.1712C>A (Ala571Asp), c.1863G>T (Trp621Cys), one synonymous variant c.1737T>C (Asp579Asp), one splice donor (c.321+2T>C), and eight intronic variants (Supplementary Table 5).

Heterozygous carriage of 4 rare missense variants, c.596G>A, c.733A>G, c.1712C>A and c.1863G>T, were reported in isolated cases of patients with severe fluoropyrimidine-related toxicities across a Japanese cohort study ^53^. Over 80% of the *in silico* prediction tools we used predicted c.596G>A, c.1712C>A, and c.1863G>T to be deleterious while c.733A>G was predicted to be benign with neutral DPYD-Varifier classification (Table 3, Supplementary Table 6). The MAF of these 4 variants are either undetermined or <0.01% in reference populations of East Asian descent (Supplementary Table 1) ^43,45^.

Eleven Korean patients from a cohort study were reported to be heterozygous carriers for the synonymous c.1737T>C variant ^46^. No allele frequency information in reference populations of East Asian descent and other ancestries has been reported for this variant (Supplementary Table 1) ^43,45^.

Tong and colleagues (2018) reported a novel intron 4 splice donor variant c.321+2T>C in a Chinese Hong Kong case study patient who developed grade 3-4 bone marrow toxicity and grade 3-4 diarrhoea to CAPEOX treatment (Supplementary Table 5). Subsequent change in chemotherapy regimen to FOLFOX was tolerated with 60% dose reduction of 5-FU. No other *DPYD* variants were identified in this patient via exome sequencing. *In silico* findings by Tong and colleagues’ (2018) suggest this variant leads to exon 4 skipping ^57^.

Of the 8 intronic variants, six were predicted to have no effect on splicing (Table 3, Supplementary Table 6). *In silico* splicing prediction for the remaining 2 intronic variants, IVS22+585C>T and IVS23-69A>G, could not be undertaken because their base pair positions could not be confirmed.

*Haplotypes*

A total of 17 *DPYD* haplotype combinations were identified in patients of East Asian ancestry with 3 in the Chinese, 5 in the Japanese, and 9 in the Korean patients (Supplementary Table 7). The normal function missense variant c.85T>C was present in all 3 haplotype combinations in the Chinese. Interestingly, the normal function missense variant c.1627A>G was present in 4 out of 5 haplotype combinations in the Japanese and 8 out of 9 haplotype combinations in the Korean. In addition to c.1627A>G, the intron 13 variant c.1740+39C>T was also present in 1 Japanese haplotype and 7 Korean haplotypes.

***Latin American***

*CPIC-reported normal DPD enzyme function*

When patients with grade 0-2 toxicities were compared to patients with grade 3-4 toxicities, a statistically significant association was observed with the variant c.85T>C (OR = 4.20, *p* = 0.002) but not with c.496A>G (OR = 2.71, *p* = 0.21) or c.1627A>G (OR = 0.45, *p* = 0.071) (Supplementary Table 5) ^58^. The MAF of these 3 variants in Admixed American reference populations are ~23%, ~6%, and ~26% respectively (Supplementary Table 1) ^43,45^.

*Haplotypes*

Four haplotype combinations were identified, with the most common haplotype identified in 9 Chilean patients consisting of c.85T>C and c.1627A>G (Supplementary Table 7).

***Middle Eastern***

Of the 13 *DPYD* variants identified, 7 are reported in the CPIC guideline whilst 6 are not present in the CPIC guideline (Supplementary Table 5).

*CPIC-reported loss of DPD enzyme function*

A Lebanese case study patient heterozygous for the c.1905+1G>A variant was identified. The patient developed grade 4 mucositis 1 week after her first cycle of FOLFIRINOX chemotherapy which led to change of chemotherapy regimen ^59^ (Supplementary Table 5). The MAF of this variant in gnomAD samples of Middle Eastern ancestry is 0.3% (Supplementary Table 1) ^45^.

*CPIC-reported decreased function*

One patient heterozygous for c.1679T>G was reported in a Tunisian cohort study and borderline significant association with hepatotoxicity (OR = 3.85, *p* = 0.048) was observed ^60^ (Supplementary Table 5). The prevalence of this variant in the gnomAD Middle Eastern population is 0% (Supplementary Table 1) ^45^.

*CPIC-reported normal function*

5 missense variants considered to have no impact on DPD enzyme function by the CPIC guideline, c.85T>C, c.496A>G, c.1601G>A (Ser534Asn), c.1627A>G, and c.2194G>A, have been identified (Supplementary Table 5). The MAF of these 5 variants in the gnomAD Middle Eastern population are ~23%, ~13%, ~3%, ~15%, and ~12%, respectively (Supplementary Table 1) ^45^.

The c.85T>C was reported in 3 cohort studies, 1 from Jordan which observed a significant association with 5-FU toxicity (OR = 0.128, *p* = 0.002) ^61^, and 2 from Tunisia where Khalij and team (2022) showed significant association with increased risk of mucositis (OR = 4.35, *p* = 0.031) and neurotoxicity (OR = 3.79, *p* = 0.029) ^60^.

The c.1601G>A variant was reported in 3 studies: one homozygous c.1601G>A variant carrier was reported in a Saudi Arabian case study ^62^; one Tunisian cohort study patient who was heterozygous for c.85T>C, c.496A>G, c.1601G>A and c.1627A>G with low plasma UH2/U ratio ^63^; and one heterozygous carrier was reported in the same Lebanese case report who was heterozygous for the splice donor c.1905+1G>A variant as described above, in addition this Lebanese patient was also a heterozygous carrier for c.2194G>A ^59^.

*Unreported in CPIC*

The missense variant c.257C>T (Pro86Leu) was reported in a Saudi Arabian case series patient ^62^. 100% of the *in silico* prediction tools we used predicted c.257C>T to be deleterious and previously published *in vitro* expression analysis reported significantly reduced DPD activity up to 97% (Table 3, Supplementary Table 6) ^64^. The nonsynonymous variant c.2434G>A (Val812Ile) was reported in another Saudi Arabian case series patient ^62^ but over 65% of the *in silico* prediction tools we used predicted this variant to be benign (Table 3, Supplementary Table 6). The MAF of c.257C>T and c.2434G>A in the gnomAD Middle Eastern population are 0% and 0.07%, respectively (Supplementary Table 1) ^45^.

We were able to perform *in silico* prediction analysis for 3 out of the 4 intronic variants reported, c.1129-15T>C, c.1740+40A>G, and c.1740+39C>T, but none were found to affect splicing (Table 3, Supplementary Table 6). The MAF of these 3 intronic variants in the gnomAD Middle Eastern population are ~15%, ~42%, and ~15%, respectively (Supplementary Table 1) ^45^. Almashagbah and team (2022) found significant associations between 5-FU toxicity and c.1740+40A>G (OR = 0.157, *p* = 0.005) and c.1740+39C>T (OR = 0.157, *p* = 0.019) in their cohort of Jordanian patients. In addition, they reported significant association with a novel insertion (g.97515583_97515584insA) (OR = 0.157, *p* = 0.017) which had a MAF of 10% in their population. We were unable to conduct *in silico* prediction for this novel insertion but the authors suggested that this variant is possibly deleterious ^61^.

*Haplotypes*

Two haplotypes were identified, one in a Lebanese case study patient and the other in a Tunisian cohort study patient. The normal function missense c.1601G>A variant was present in both haplotypes (Supplementary Table 7).

***South Asian***

Of the 7 *DPYD* variants reported, 6 except 1 are reported in the CPIC guideline (Supplementary Table 5).

*CPIC-reported normal function*

The nonsynonymous variants c.85T>C, c.496A>G, c.1601G>A, c.1627A>G and c.2194G>A were reported in Indian patients from 5 cohort studies, a case series, and a case report, ^65-70^. The MAF of c.85T>C, c.496A>G, c.1627A>G and c.2194G>A in reference populations of South Asian descent are approximately 26%, 7%, 8.5%, and 9%, respectively (Supplementary Table 1) ^43,45^. c.1601G>A however is rare and has a MAF of 0.5-0.9% in reference populations of South Asian descent (Supplementary Table 1) ^43,45^.

A heterozygous c.1601G>A patient was reported in an Indian cohort study ^67^. Significant decrease in the incidence of mucositis (*p* = 0.0198; *p* = 0.016) and diarrhoea (*p* = 0.0325; *p* = 0.006) were reported in 2 of the 5 Indian cohort studies after 50% dose reduction of fluoropyrimidine in cycle 2 of chemotherapy in patients carrying *DPYD* variants including c.85T>C, c.496A>G, c.1601G>A, c.1627A>G, c.1905+1G>A, c.2194G>A, and/or c.2846A>T ^67,69^.

*Unreported in CPIC*

Heterozygous carriage of the exon 7 missense variant c.704G>A (Arg235Gln) was reported in an Indian patient from an American case report ^71^. This variant is not included in the current CPIC guideline but was predicted to be deleterious by 100% of the *in silico* prediction tools we used and was classified as deleterious by the DPYD-Varifier (Table 3, Supplementary Table 6). The prevalence of c.704G>A is rare and has a prevalence of 0.002% in the gnomAD v4.0.0 South Asian reference population (Supplementary Table 1) ^45^.

*Haplotypes*

Five haplotypes were identified with 3 in 5 haplotype combinations containing either c.496A>G or c.2194G>A (Supplementary Table 7).

**Supplementary Discussion**

It is important to briefly consider some other variants. First, c.464T>A (p.Leu155Ter, rs2101026231), a nonsense variant, located on exon 5, causes the replacement of leucine 155 by a stopping codon, resulting in a truncated protein. This variant was first reported in a Spanish patient who died from severe, multi-system toxicity following the first administration of 5-FU for adjuvant colon cancer therapy ^72^. This variant was also reported in two Chinese patients with 5-FU-related severe toxicity who both exhibited ~45% lower DPD enzyme activity in PBMCs compared to non-carriers ^54^. This variant is not included in the current CPIC guideline and its allele frequency across global ethnic populations in the 1000 Genomes and gnomAD databases is currently unknown, but our *in silico* analysis predicted this variant to be deleterious but further *in vitro* functional work is required to confirm the impact of this variant on DPD enzyme activity. Second, the exon 11 truncating variant, c.1156G>T (p.Glu386Ter, rs78060119, *12), leads to premature protein truncation at amino acid position 386 and is classified as a loss-of-function variant with moderate evidence level by the CPIC guideline. Heterozygous carriage of this variant was detected in three Japanese patients with severe fluoropyrimidine-related toxicity across 2 case studies and 1 cohort study identified in our systematic review ^53,55,73^. *In vivo* and *in vitro* studies of this variant observed over 90% reduction in DPD activity ^42,55,73^. Currently, there is no guideline or mandate for *DPYD* testing before fluoropyrimidine treatment in Japan but *DPYD* genetic testing that includes the c.1156G>T variant is available in several hospital pharmacies and can be requested by the attending physician. Data from the latest gnomAD release (v4.0) showed very low prevalence (<0.005%) of this variant across East Asian, Admixed American, South Asian, and European populations. Third, the c.1774C>T (p.Arg592Trp, rs59086055) variant has a prevalence of 0.1% in East Asian reference populations ^43,45^; heterozygous carriage of c.1774C>T was detected in 1 Korean patient ^46^ and 1 Thai patient ^51^ in our systematic review. This exon 14 missense variant causing Arg592Trp substitution is considered a loss-of-function variant by the CPIC guideline with weak evidence. No *in vivo* data have been published for this variant but *in vitro* functional work reported over 90% reduction in DPD activity ^42,74-76^. These variants seem to be important but further work is required both to understand the functional relevance of these variants, and identify other variants in East Asian individuals, to improve the prediction of fluoropyrimidine-related toxicity in the different ethnic groups that constitute East Asian populations in the UK and globally.

**References**

1. Ng PC, Henikoff S. Predicting deleterious amino acid substitutions. Genome Res. 2001;11:863-874.

2. Ng PC, Henikoff S. SIFT: Predicting amino acid changes that affect protein function. Nucleic Acids Res. 2003;31:3812-3814.

3. Adzhubei IA, Schmidt S, Peshkin L, Ramensky VE, Gerasimova A, Bork P *et al.* A method and server for predicting damaging missense mutations. Nat Methods. 2010;7:248-249.

4. Adzhubei I, Jordan DM, Sunyaev SR. Predicting functional effect of human missense mutations using PolyPhen-2. Curr Protoc Hum Genet. 2013;76:7.20.21-27.20.41.

5. Pejaver V, Urresti J, Lugo-Martinez J, Pagel KA, Lin GN, Nam HJ *et al.* Inferring the molecular and phenotypic impact of amino acid variants with MutPred2. Nat Commun. 2020;11:5918.

6. Li B, Krishnan VG, Mort ME, Xin F, Kamati KK, Cooper DN *et al.* Automated inference of molecular mechanisms of disease from amino acid substitutions. Bioinformatics. 2009;25:2744-2750.

7. Jagadeesh KA, Wenger AM, Berger MJ, Guturu H, Stenson PD, Cooper DN *et al.* M-CAP eliminates a majority of variants of uncertain significance in clinical exomes at high sensitivity. Nat Genet. 2016;48:1581-1586.

8. Steinhaus R, Proft S, Schuelke M, Cooper DN, Schwarz JM, Seelow D. MutationTaster2021. Nucleic Acids Res. 2021;49:W446-W451.

9. Kircher M, Witten DM, Jain P, O'Roak BJ, Cooper GM, Shendure J. A general framework for estimating the relative pathogenicity of human genetic variants. Nat Genet. 2014;46:310-315.

10. Rentzsch P, Witten D, Cooper GM, Shendure J, Kircher M. CADD: predicting the deleteriousness of variants throughout the human genome. Nucleic Acids Res. 2019;47:D886-D894.

11. Rentzsch P, Schubach M, Shendure J, Kircher M. CADD-Splice-improving genome-wide variant effect prediction using deep learning-derived splice scores. Genome Med. 2021;13:31.

12. Carter H, Douville C, Stenson PD, Cooper DN, Karchin R. Identifying Mendelian disease genes with the variant effect scoring tool. BMC Genomics. 2013;14:S3.

13. Douville C, Carter H, Kim R, Niknafs N, Diekhans M, Stenson PD *et al.* CRAVAT: cancer-related analysis of variants toolkit. Bioinformatics. 2013;29:647-648.

14. Masica DL, Douville C, Tokheim C, Bhattacharya R, Kim R, Moad K *et al.* CRAVAT 4: Cancer-Related Analysis of Variants Toolkit. Cancer Res. 2017;77:e35-e38.

15. Douville C, Masica DL, Stenson PD, Cooper DN, Gygax DM, Kim R *et al.* Assessing the Pathogenicity of Insertion and Deletion Variants with the Variant Effect Scoring Tool (VEST-Indel). Hum Mutat. 2016;37:28-35.

16. Ioannidis NM, Rothstein JH, Pejaver V, Middha S, McDonnell SK, Baheti S *et al.* REVEL: An Ensemble Method for Predicting the Pathogenicity of Rare Missense Variants. Am J Hum Genet. 2016;99:877-885.

17. Reva B, Antipin Y, Sander C. Determinants of protein function revealed by combinatorial entropy optimization. Genome Biol. 2007;8:R232.

18. Reva B, Antipin Y, Sander C. Predicting the functional impact of protein mutations: application to cancer genomics. Nucleic Acids Research. 2011;39:e118-e118.

19. Niroula A, Vihinen M. How good are pathogenicity predictors in detecting benign variants? PLoS Comput Biol. 2019;15:e1006481.

20. Dong C, Wei P, Jian X, Gibbs R, Boerwinkle E, Wang K *et al.* Comparison and integration of deleteriousness prediction methods for nonsynonymous SNVs in whole exome sequencing studies. Hum Mol Genet. 2015;24:2125-2137.

21. Shihab HA, Gough J, Mort M, Cooper DN, Day IN, Gaunt TR. Ranking non-synonymous single nucleotide polymorphisms based on disease concepts. Hum Genomics. 2014;8:11.

22. Shihab HA, Gough J, Cooper DN, Stenson PD, Barker GL, Edwards KJ *et al.* Predicting the functional, molecular, and phenotypic consequences of amino acid substitutions using hidden Markov models. Hum Mutat. 2013;34:57-65.

23. Shihab HA, Gough J, Cooper DN, Day IN, Gaunt TR. Predicting the functional consequences of cancer-associated amino acid substitutions. Bioinformatics. 2013;29:1504-1510.

24. Bendl J, Musil M, Stourac J, Zendulka J, Damborsky J, Brezovsky J. PredictSNP2: A Unified Platform for Accurately Evaluating SNP Effects by Exploiting the Different Characteristics of Variants in Distinct Genomic Regions. PLoS Comput Biol. 2016;12:e1004962.

25. Jaganathan K, Kyriazopoulou Panagiotopoulou S, McRae JF, Darbandi SF, Knowles D, Li YI *et al.* Predicting Splicing from Primary Sequence with Deep Learning. Cell. 2019;176:535-548 e524.

26. Desmet FO, Hamroun D, Lalande M, Collod-Beroud G, Claustres M, Beroud C. Human Splicing Finder: an online bioinformatics tool to predict splicing signals. Nucleic Acids Res. 2009;37:e67.

27. Reese MG, Eeckman FH, Kulp D, Haussler D. Improved splice site detection in Genie. J Comput Biol. 1997;4:311-323.

28. Zuallaert J, Godin F, Kim M, Soete A, Saeys Y, De Neve W. SpliceRover: interpretable convolutional neural networks for improved splice site prediction. Bioinformatics. 2018;34:4180-4188.

29. Messeguer X, Escudero R, Farre D, Nunez O, Martinez J, Alba MM. PROMO: detection of known transcription regulatory elements using species-tailored searches. Bioinformatics. 2002;18:333-334.

30. Farre D, Roset R, Huerta M, Adsuara JE, Rosello L, Alba MM *et al.* Identification of patterns in biological sequences at the ALGGEN server: PROMO and MALGEN. Nucleic Acids Res. 2003;31:3651-3653.

31. Kumar S, Ambrosini G, Bucher P. SNP2TFBS - a database of regulatory SNPs affecting predicted transcription factor binding site affinity. Nucleic Acids Res. 2017;45:D139-D144.

32. Moradifard S, Saghiri R, Ehsani P, Mirkhani F, Ebrahimi-Rad M. A preliminary computational outputs versus experimental results: Application of sTRAP, a biophysical tool for the analysis of SNPs of transcription factor-binding sites. Mol Genet Genomic Med. 2020;8:e1219.

33. Ziebarth JD, Bhattacharya A, Chen A, Cui Y. PolymiRTS Database 2.0: linking polymorphisms in microRNA target sites with human diseases and complex traits. Nucleic Acids Res. 2012;40:D216-221.

34. Bhattacharya A, Ziebarth JD, Cui Y. PolymiRTS Database 3.0: linking polymorphisms in microRNAs and their target sites with human diseases and biological pathways. Nucleic Acids Res. 2014;42:D86-91.

35. Barenboim M, Zoltick BJ, Guo Y, Weinberger DR. MicroSNiPer: a web tool for prediction of SNP effects on putative microRNA targets. Hum Mutat. 2010;31:1223-1232.

36. Shrestha S, Zhang C, Jerde CR, Nie Q, Li H, Offer SM *et al.* Gene-Specific Variant Classifier (DPYD-Varifier) to Identify Deleterious Alleles of Dihydropyrimidine Dehydrogenase. Clin Pharmacol Ther. 2018;104:709-718.

37. Zhou Y, Dagli Hernandez C, Lauschke VM. Population-scale predictions of DPD and TPMT phenotypes using a quantitative pharmacogene-specific ensemble classifier. Br J Cancer. 2020;123:1782-1789.

38. Zhou Y, Mkrtchian S, Kumondai M, Hiratsuka M, Lauschke VM. An optimized prediction framework to assess the functional impact of pharmacogenetic variants. Pharmacogenomics J. 2019;19:115-126.

39. Saif MW, Lee AM, Offer SM, McConnell K, Relias V, Diasio RB. A DPYD variant (Y186C) specific to individuals of African descent in a patient with life-threatening 5-FU toxic effects: potential for an individualized medicine approach. Mayo Clin Proc. 2014;89:131-136.

40. Sissung TM, Cordes L, Peer CJ, Gandhy S, Redman J, Strauss J *et al.* Case report: severe toxicity in an African-American patient receiving FOLFOX carrying uncommon allelic variants in DPYD. Pharmacogenomics. 2021;22:81-85.

41. Chen S, Francioli LC, Goodrich JK, Collins RL, Kanai M, Wang Q *et al.* A genomic mutational constraint map using variation in 76,156 human genomes. Nature. 2024;625:92-100.

42. Offer SM, Fossum CC, Wegner NJ, Stuflesser AJ, Butterfield GL, Diasio RB. Comparative functional analysis of DPYD variants of potential clinical relevance to dihydropyrimidine dehydrogenase activity. Cancer Res. 2014;74:2545-2554.

43. Genomes Project C, Auton A, Brooks LD, Durbin RM, Garrison EP, Kang HM *et al.* A global reference for human genetic variation. Nature. 2015;526:68-74.

44. Maharjan AS, McMillin GA, Patel GK, Awan S, Taylor WR, Pai S *et al.* The Prevalence of DPYD*9A(c.85T>C) Genotype and the Genotype-Phenotype Correlation in Patients with Gastrointestinal Malignancies Treated With Fluoropyrimidines: Updated Analysis. Clin Colorectal Cancer. 2019;18:e280-e286.

45. Chen S, Francioli L, Goodrich J, Collins R, Kanai M, Wang Q *et al.* A genome-wide mutational constraint map quantified from variation in 76,156 human genomes. 2022.

46. Cho HJ, Park YS, Kang WK, Kim JW, Lee SY. Thymidylate synthase (TYMS) and dihydropyrimidine dehydrogenase (DPYD) polymorphisms in the Korean population for prediction of 5-fluorouracil-associated toxicity. Ther Drug Monit. 2007;29:190-196.

47. Deng X, Hou J, Deng Q, Zhong Z. Predictive value of clinical toxicities of chemotherapy with fluoropyrimidines and oxaliplatin in colorectal cancer by DPYD and GSTP1 gene polymorphisms. World J Surg Oncol. 2020;18:321.

48. Kanai M, Kawaguchi T, Kotaka M, Manaka D, Hasegawa J, Takagane A *et al.* Poor association between dihydropyrimidine dehydrogenase (DPYD) genotype and fluoropyrimidine-induced toxicity in an Asian population. Cancer Med. 2023;12:7808-7814.

49. Liu D, Li J, Gao J, Li Y, Yang R, Shen L. Examination of multiple UGT1A and DPYD polymorphisms has limited ability to predict the toxicity and efficacy of metastatic colorectal cancer treated with irinotecan-based chemotherapy: a retrospective analysis. BMC Cancer. 2017;17:437.

50. Nie QH, Guo XQ, Liu HF, Zeng L, Wang X, Wen SL *et al.* Effects of DPYD and TS gene polymorphisms on chemosensitivity of 5-FU in advanced colorectal cancer. Int J Clin Exp Med. 2019;12:9380-9386.

51. Sirachainan E, Reungwetwattana T, Wisetpanit Y, Panvichian R, Sirisinha T, Ativitavas T *et al.* Pharmacogenetic Study of 5-Fluorouracil-Related Severe Toxicity in Thai Cancer Patients: A Novel SNP Detection. Journal of Pharmacogenomics & Pharmacoproteomics. 2012;3:1-4.

52. Sun W, Yan C, Jia S, Hu J. Correlation analysis of peripheral DPYD gene polymorphism with 5-fluorouracil susceptibility and side effects in colon cancer patients. Int J Clin Exp Med. 2014;7:5857-5861.

53. Yokoi K, Nakajima Y, Matsuoka H, Shinkai Y, Ishihara T, Maeda Y *et al.* Impact of DPYD, DPYS, and UPB1 gene variations on severe drug-related toxicity in patients with cancer. Cancer Sci. 2020;111:3359-3366.

54. Zhang X, Sun B, Lu Z. Evaluation of clinical value of single nucleotide polymorphisms of dihydropyrimidine dehydrogenase gene to predict 5-fluorouracil toxicity in 60 colorectal cancer patients in China. Int J Med Sci. 2013;10:894-902.

55. Kouwaki M, Hamajima N, Sumi S, Nonaka M, Sasaki M, Dobashi K *et al.* Identification of novel mutations in the dihydropyrimidine dehydrogenase gene in a Japanese patient with 5-fluorouracil toxicity. Clin Cancer Res. 1998;4:2999-3004.

56. Ishiguro M, Takenaka R, Ogura K, Hiratsuka A, Takeda H, Kawai D *et al.* A Japanese Patient with Gastric Cancer and Dihydropyrimidine Dehydrogenase Deficiency Presenting with DPYD Variants. Acta Med Okayama. 2020;74:557-562.

57. Tong CC, Lam CW, Lam KO, Lee VHF, Luk MY. A Novel DPYD Variant Associated With Severe Toxicity of Fluoropyrimidines: Role of Pre-emptive DPYD Genotype Screening. Front Oncol. 2018;8:279.

58. Cordova-Delgado M, Bravo ML, Cumsille E, Hill CN, Munoz-Medel M, Pinto MP *et al.* A case-control study of a combination of single nucleotide polymorphisms and clinical parameters to predict clinically relevant toxicity associated with fluoropyrimidine and platinum-based chemotherapy in gastric cancer. BMC Cancer. 2021;21:1030.

59. Mukherji D, Massih SA, Tfayli A, Kanso M, Faraj W. Three different polymorphisms of the DPYD gene associated with severe toxicity following administration of 5-FU: a case report. J Med Case Rep. 2019;13:76.

60. Khalij Y, Belaid I, Chouchane S, Amor D, Omezzine A, Ben Rejeb N *et al.* DPYD and TYMS polymorphisms as predictors of 5 fluorouracil toxicity in colorectal cancer patients. J Chemother. 2023;35:425-434.

61. Almashagbah NA, Mahasneh AA, Bodoor KG. Pharmacogenetic Study of the Dihydropyridine Dehydrogenase Gene in Jordanian Patients with Colorectal Cancer. Asian Pac J Cancer Prev. 2022;23:3061-3069.

62. Bukhari N, Alshangiti A, Tashkandi E, Algarni M, Al-Shamsi HO, Al-Khallaf H. Fluoropyrimidine-Induced Severe Toxicities Associated with Rare DPYD Polymorphisms: Case Series from Saudi Arabia and a Review of the Literature. Clin Pract. 2021;11:467-471.

63. Ben Fredj R, Gross E, Ben Ahmed S, Hassine H, Saguem S. The dihydrouracil/uracil ratio in plasma, clinical and genetic analysis for screening of dihydropyrimidine dehydrogenase deficiency in colorectal cancer patients treated with 5-fluorouracil. Pathol Biol (Paris). 2009;57:470-476.

64. Elraiyah T, Jerde CR, Shrestha S, Wu R, Nie Q, Giama NH *et al.* Novel Deleterious Dihydropyrimidine Dehydrogenase Variants May Contribute to 5-Fluorouracil Sensitivity in an East African Population. Clin Pharmacol Ther. 2017;101:382-390.

65. Dhawan D, Panchal H, Shukla S, Padh H. Genetic variability & chemotoxicity of 5-fluorouracil & cisplatin in head & neck cancer patients: a preliminary study. Indian J Med Res. 2013;137:125-129.

66. Hariprakash JM, Vellarikkal SK, Keechilat P, Verma A, Jayarajan R, Dixit V *et al.* Pharmacogenetic landscape of DPYD variants in south Asian populations by integration of genome-scale data. Pharmacogenomics. 2018;19:227-241.

67. Patil VM, Noronha V, Joshi A, Zanwar S, Ramaswamy A, Arya S *et al.* Dihydropyrimidine dehydrogenase mutation in neoadjuvant chemotherapy in head and neck cancers: Myth or reality? South Asian J Cancer. 2016;5:182-185.

68. Rastogi S, Sirohi B, Deodhar K, Shetty N, Shrikhande SV. Dilemma of dihydropyrimidine dehydrogenase deficiency in colorectal cancer patients: is Uftoral(R) the right answer? Colorectal Cancer. 2014;3:315-319.

69. Sahu A, Ramaswamy A, Ostwal V. Dihydro pyrimidine dehydrogenase deficiency in patients treated with capecitabine based regimens: a tertiary care centre experience. J Gastrointest Oncol. 2016;7:380-386.

70. Vinin NV, Jones J, Geetha M. Clinical Suspicion & Dpd/Dypd Mutation Positivity In Patients Receiving Chemotherapy With Capecitabine / 5 Fluorouracil (5 Fu). Journal of Cancer Research & Therapeutics. 2017;13:S218-S218.

71. Ly RC, Schmidt RE, Kiel PJ, Pratt VM, Schneider BP, Radovich M *et al.* Severe Capecitabine Toxicity Associated With a Rare DPYD Variant Identified Through Whole-Genome Sequencing. JCO Precis Oncol. 2020;4:632-638.

72. Morel A, Boisdron-Celle M, Fey L, Laine-Cessac P, Gamelin E. Identification of a novel mutation in the dihydropyrimidine dehydrogenase gene in a patient with a lethal outcome following 5-fluorouracil administration and the determination of its frequency in a population of 500 patients with colorectal carcinoma. Clin Biochem. 2007;40:11-17.

73. Yoshida Y, Ogura K, Hiratsuka A, Aisu N, Yamada T, Kojima D *et al.* 5-Fluorouracil Chemotherapy for Dihydropyrimidine Dehydrogenase-deficient Patients: Potential of the Dose-escalation Method. Anticancer Res. 2015;35:4881-4887.

74. Hishinuma E, Gutierrez Rico E, Hiratsuka M. In Vitro Assessment of Fluoropyrimidine-Metabolizing Enzymes: Dihydropyrimidine Dehydrogenase, Dihydropyrimidinase, and beta-Ureidopropionase. J Clin Med. 2020;9:2342.

75. Hishinuma E, Narita Y, Saito S, Maekawa M, Akai F, Nakanishi Y *et al.* Functional Characterization of 21 Allelic Variants of Dihydropyrimidine Dehydrogenase Identified in 1070 Japanese Individuals. Drug Metab Dispos. 2018;46:1083-1090.

76. Hishinuma E, Narita Y, Obuchi K, Ueda A, Saito S, Tadaka S *et al.* Importance of Rare DPYD Genetic Polymorphisms for 5-Fluorouracil Therapy in the Japanese Population. Front Pharmacol. 2022;13:930470.

**Supplementary Table Legends**

**Supplementary Table 1: Minor allele frequencies of the four prominent European *DPYD* variants and all other *DPYD* variants evaluated in our systematic review.**

**Supplementary Table 2: MEDLINE (PubMed) search strategy.**

**Supplementary Table 3: List of in silico tools utilised to assess *DPYD* variants evaluated in our systematic review.**

**Supplementary Table 4: Summary characteristics of included articles.**

**Supplementary Table 5: Details of all extracted data items including genotype counts of *DPYD* variants reported in non-European patients with severe fluoropyrimidine-related toxicity.**

**Supplementary Table 6: Detailed *in silico* predictions and *in vitro* analyses of *DPYD* variants evaluated in our systematic review.**

**Supplementary Table 7: Potential/partial *DPYD* haplotypes identified in our systematic review.**
